# Systematic Review of Workplace Interventions to Support Young Workers’ Safety, Work Environment and Health

**DOI:** 10.1007/s10926-024-10186-y

**Published:** 2024-04-30

**Authors:** Emil Sundstrup, Karina Glies Vincents Seeberg, Johnny Dyreborg, Thomas Clausen, Lars Louis Andersen

**Affiliations:** 1 National Research Centre for the Working Environment, Lersø Parkallé 105, 2100 Copenhagen, Denmark; 2 Department of Health Science and Technology, Aalborg University, 9220 Aalborg, Denmark

**Keywords:** Workers, Mental health, Stress, Musculoskeletal disorders, Injuries, Occupational

## Abstract

**Purpose:**

This systematic review investigates the effectiveness of workplace interventions to support young workers’ work environment, safety and health.

**Methods:**

A systematic search was conducted in bibliographic databases including PubMed, Web of Science Core Collection and PsycInfo for English or Scandinavian articles published from 2007 to 2022. The PICO strategy guided the assessment of study relevance and the bibliographical search for randomized controlled trials (RCTs) and non-RCTs in which (1) participants were young workers (mean age: 15–29), (2) interventions were initiated and/or carried out at the workplace, (3) a comparison group was included, and (4) an outcome measure related to work environment, safety and health was reported. We categorized each included study using the intervention classification framework. The quality assessment and evidence synthesis adhered to the guidelines developed by the Institute for Work & Health (Toronto, Canada).

**Results:**

A total of 33 high and medium quality studies showed a moderate level of evidence for no benefit of ‘Mental training’ on stress. We found limited evidence of a positive effect of the following intervention types: ‘Attitude and belief’ on mental health problems, ‘Behavior based’ on anxiety, and ‘Multifaceted’ on hand eczema. We found limited evidence for no benefit of the following intervention types: ‘Mental training’ on mental health problems, and ‘Physiological modifications’ on musculoskeletal disorders. The remaining intervention types showed mixed or insufficient evidence.

**Conclusions:**

Except for a moderate level of evidence for no benefit of ‘Mental training’ on stress, the evidence synthesis recommends, that there is not enough evidence from the scientific literature to guide current practices. The results emphasizes a strong need for high quality interventions specifically aiming at increasing or maintaining young workers’ work environment, safety and health. Included studies focused mainly on individual measures, highlighting the need for studies investigating possible preventive measures at the group or organizational level.

**Supplementary Information:**

The online version contains supplementary material available at 10.1007/s10926-024-10186-y.

## Background

Young workers are particularly vulnerable in relation to accidents at work and injuries at their workplace, and thus face potentially harmful exposures and hazardous work to a greater degree than older workers [1–3]. Recent literature shows that young workers report a higher degree of physically demanding work than older workers, as well as more symptoms of depression and anxiety [1–3]. Young workers, crucial to both private and public companies, should have a healthy and safe start in working life to ensure their longevity in the labor market until retirement. Knowledge about effective workplace interventions is needed to secure a sustainable working life and reduce the burden of accidents at work, musculoskeletal disorders and illnesses, and mental health problems for young workers.

Previous research has shown that young workers are more likely to experience serious accidents at work than their older colleagues [1, 4, 5]. A recent report summarizing the work environment and health among young workers in Denmark revealed that a larger proportion of young workers consider accidents as a normal part of their daily work, highlighting the potential to improve safety culture and occupational health and safety competence [2]. Young workers also experience higher levels of physically demanding work tasks, such as heavy lifting and working in awkward postures, compared to their older colleagues [1, 2] which are well-known risk factors for the development of musculoskeletal disorders (MSDs) and premature exit from the labour market [1, 6–9]. Further, they report higher levels of exposure to chemical substances which has been associated with skin reactions, e.g., hand eczema [1, 2]. In the context of accidents at work, the consequences are immediately apparent as acute occupational injuries. By contrast, most occupational diseases, such as MSDs and mental health problems, develop due to accumulative exposure often over years, emphasizing the importance of preventive measures starting when entering the working life. Given that occupational diseases typically evolve over several years due to cumulative risk exposure, the increased vulnerability of young workers further emphasizes the critical need for early preventive measures.

A large cohort study on young workers in Denmark also revealed that young workers report higher levels of depressive symptoms and anxiety compared to their older counterparts [2], which also constitutes a risk factor for a premature exit from the labour market [10]. Previous studies have shown that psychosocial work factors, such as high job demands, increase the risk of mental health problems among young workers [1, 11]. Further, a recent study on more than 300.000 young employees in Denmark found that several psychosocial work factors were associated with sickness absence spells of any length (≥ 1 day) [11]. Specifically, they found that among women, employment in occupations with high quantitative demands, low decision authority, high job strain, high emotional demands, or high work-related physical violence was associated with higher rates of sickness absence. On the other hand, Andersen et al. found that younger workers may be more resistant to high physical workloads than older workers in terms of sickness absence risk [12]. Thus, while definitive knowledge is still needed, the above challenges have resulted in increased awareness of occupational health and safety of young workers worldwide with a focus on securing a sustainable working life for this group of workers.

Recent years of research and mapping of young people’s working environment have provided a necessary basis for effectively targeting efforts aimed at relevant industries and groups of young people who are particularly vulnerable. Currently, there are no systematic reviews documenting and summarizing the scientific literature on the effect of workplace interventions to support young workers’ safety, work environment and health. A previous review by Hanvold et al. aimed to identify risk factors for occupational accidents and illnesses among young workers and to attain knowledge on specific vulnerable groups of young workers—also from intervention studies [1]. That study emphasized, that there is a lack of scientific studies and knowledge concerning the effects of preventive actions regarding young workers and accidents at work in the Nordic countries. The authors further stated that there is a need for more studies on accidents at work and work-related health among young workers, assessing the efficiency of different interventions in increasing young workers’ occupational safety and health—and including the global literature. The novelty of this systematic review lies in addressing this knowledge gap by identifying effective measures for enhancing the working environment of young workers, who are particularly vulnerable during their first time in a new job and where special attention and preventive efforts are needed [13].

Several studies have documented that young workers represent a diverse group, engaged in various types of work with distinct occupational exposures and varying levels of attachment to the labour market [1, 2, 14, 15]. On one hand, young workers perform many different types of work with different risk conditions. On the other hand, the approach of young individuals to work can vary considerably, depending on the context of their employment—whether it is part of their vocational training, a job undertaken during their student years, or employment pursued after graduation. Further, different industries and workplaces are likely to exhibit diverse working conditions and working cultures, implying that the effectiveness of interventions for young workers can vary significantly. This emphasizes the necessity of nuanced approaches to enhancing the working environment for young people in the workforce. Such approaches should consider, on one hand, the diverse life situations of young people and their transition from education to work, and on the other hand, the specific conditions that young people encounter at the workplaces [15]. In this review, we extract all relevant existing knowledge on workplace interventions for young workers (irrespective of education, industry, positions in the labour market etc.) aiming at improving the work environment, health and safety and developing practical messages for stakeholders. To ensure the inclusion of young workers with longer educations, and thus typically entering work life in their late twenties, we include intervention studies of young workers up to the age of 29 years. This approach has previously been employed in both Danish [2] and international studies [1].

**Fig. 1:**
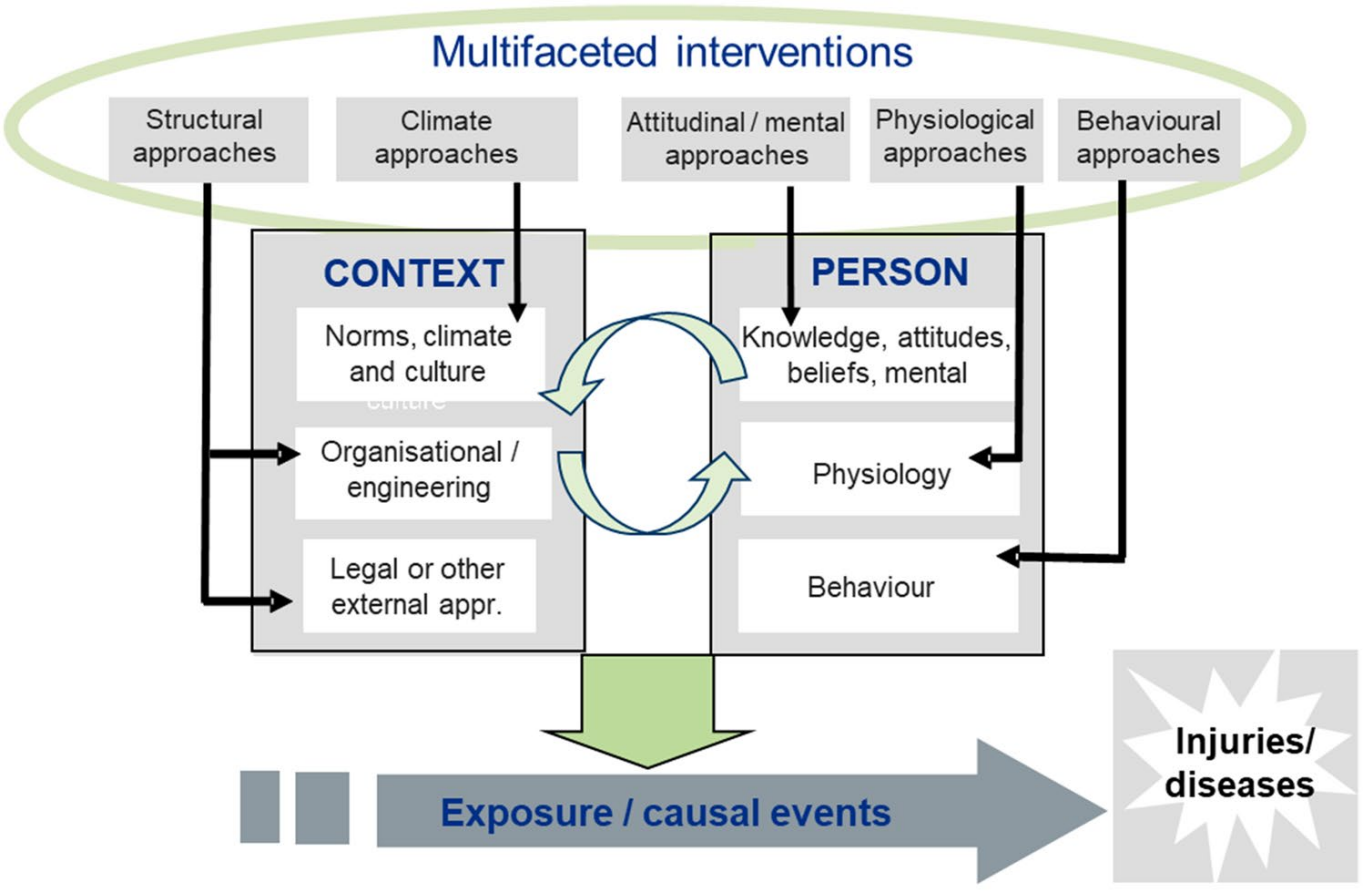
Possible pathways for promoting health and safety at work and decreasing frequency or severity of injuries and occupational diseases to people at work (logic model)

This systematic review aims to identify various types of workplace interventions targeted at young workers and to investigate their effectiveness in supporting young workers’ safety, work environment and health. To capture these various approaches to workplace interventions, and to distinguish between the various approaches, we use a logic model (Fig. 1) for the possible pathways for preventing injuries and occupational diseases related to ergonomic exposure, psychosocial exposures, chemical exposures, and risk of accidents, based on Dyreborg et al. [16]. This model provides an overall framework, with sub‐categories representing more specific types of occupational health and safety (OHS) interventions (see the methods section “Defining and coding included interventions”). These specific types of OHS interventions have been the subject of analysis in this review. The framework was initially used for different types of intervention directed at the prevention of accidents at work, but has been adjusted to include the various OHS problems among young workers included in this review. Following this, we have included the following types of approaches to preventing injuries and occupational diseases: (1) external OHS interventions, such as legislation and enforcement; (2) the organizational and physical environment at the workplace, such as engineering and safety management; (3) approaches directed at modifying social norms, climate and culture at the group or organizational level; (4) approaches directed at the behaviours of members of the organization, such as training, feedback and individual coaching; and (5) attitudes, beliefs and mental training approaches, such as campaigns and persuasive messages, posters and mental training and mindfulness. Guidance on the use of this framework is presented in the methods section “Defining and coding included interventions”.

## Methods

### Study Design and Registration

We followed the ‘Preferred Reporting Items for Systematic Reviews and Meta-Analyses’ (PRISMA) guidelines [17, 18] and the Institute for Work & Health (IWH) guidelines for workplace-based interventions in conducting this systematic review [19, 20]. To ensure the practical relevance of the review, we engaged with stakeholders representing four industry communities related to the work environment in Denmark: ‘Retail and Trade’, ‘Restaurants and Bars’, ‘Building and Construction’, and ‘Residential institutions and Home Care’. These communities, comprising representatives from employers and employees’ labour organizations, provide guidance on the working environment through guidelines, conferences, and education. This process was inspired by the IWH systematic review programme [21]. Our stakeholders were involved in the study’s conception by contributing with input to the preparation of the research application, ensuring that the topic was practical and relevant to them. When funding for the study was obtained, we held a meeting with the stakeholders to discuss and finalize the research question and provide practical input to the search strategy. This approach ensured that the literature search was comprehensive. We also gave the stakeholders an introduction to the systematic review steps and evidence synthesis methodology, aiming to increase their capacity for understanding research and thereby enhance research use in decision-making in the sectoral and jurisdictional context as needed [22, 23]. After the forming of the evidence synthesis, the results were presented for the stakeholders and they provided input to the recommendations and the dissemination of the results.

The review has been registered in the International Prospective Register of Systematic Reviews (PROSPERO; CRD42022324299): https://www.crd.york.ac.uk/prospero/display_record.php?ID=CRD42022324299

### Eligibility Criteria

Table 1 displays the eligibility criteria based on the PICO strategy used in the review. This strategy guided the assessment of study relevance and the bibliographical search for studies that focused on workers or the transition to work. The inclusion criteria were as follows: (1) participants were young workers with a mean age of 15 to 29 years, (2) interventions were workplace-based, (3) a comparison group was included, and (4) an outcome measure related to work environment, safety, and health was reported, including occupational injuries/accidents at work, musculoskeletal disorders, mental health problems, ergonomic work factors, psychosocial work factors, and safety climate. Both randomized controlled trials (RCTs) and trials that are not randomized (e.g., non-RCTs and controlled before and after studies) were eligible for inclusion, and the publication language of included studies was English or Scandinavian.

**Table 1:** Illustration of the PICO used for the present review

P	Population	Young workers (mean age: 15–29 years)
I	Intervention	Initiated by the workplace, supported by the workplace and/or carried out at the workplace (i.e. workplace-based)
C	Comparison	A comparison group was included (i.e. no treatment, treatment as usual, or another comparison treatment at the workplace)
O	Outcome	Any outcome related to work environment, safety and health, including occupational injuries/accidents, musculoskeletal disorders, mental health problems, ergonomic work factors, psychosocial work factors, and safety climate

The review focuses on primary OHS interventions that deliberately aim to support safety, work environment, and health and reduce the frequency or severity of injuries or well-being at work [24]. Therefore, only papers reporting an intervention directed at the workplace (i.e., workplace-based interventions) are included. An intervention could be initiated at the workplace by the employer, employees, or externally by public authorities, social partners, or other stakeholders. The review excluded tertiary interventions, such as return-to-work programs. Public health and safety campaigns directed at the general population and community-based health and safety interventions were also excluded from the review, as they were not primarily implemented at workplaces.

### Search Strategy

The systematic search was conducted across multiple bibliographic databases, including PubMed (including the database ‘MEDLINE’), Web of Science Core Collection (including the databases ‘Science Citation Index Expanded’, ‘Social Sciences Citation Index’, and ‘Arts & Humanities Citation Index’), and PsycInfo via OVID. To perform the search, the following three main components were combined: (1) young workers AND (2) workplace intervention AND (3) date (published from 2007 to 2022). The search strategy for each database can be found in Supplementary Material 1. To ensure a comprehensive search, manual searches were also conducted through (1) examination of reference lists, a process denoted as snowballing, of pertinent studies and (2) identification of relevant articles through personal knowledge and contacts. The manual search led to the inclusion of one study [25] that initially eluded identification through our search strategy. Before implementing the final search strategy, we conducted pilot testing with various inclusion and exclusion criteria across multiple search strings. This iterative process aimed to enhance the precision of the search strategy and, consequently, the relevance of the search outcomes within the context of our systematic review.

### Assessment of Relevance and Inclusion

**Fig. 2:**
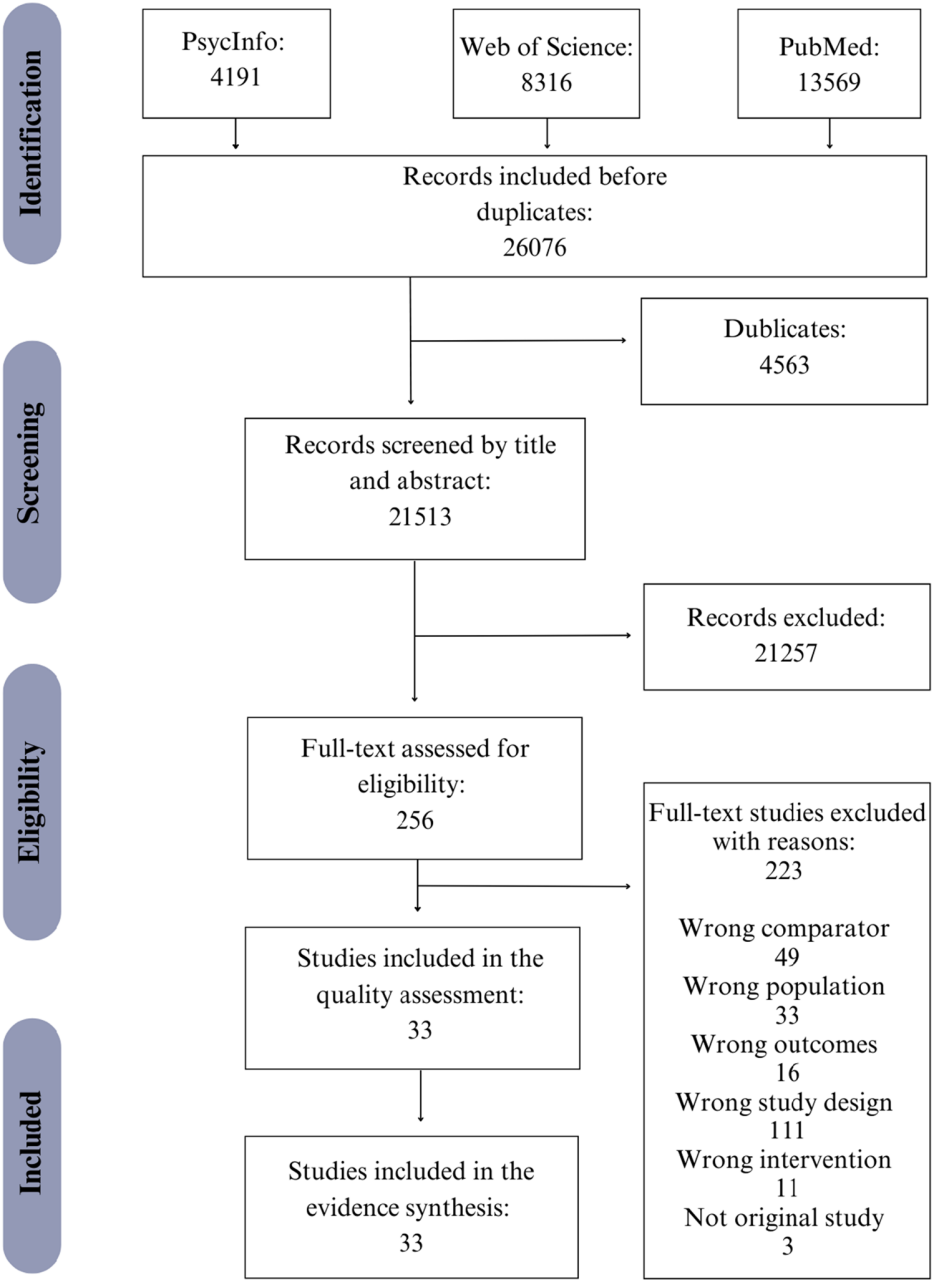
Flow chart

The study selection process is summarised in the PRISMA flow diagram presented in Fig. 2. The search for potential studies was conducted using EndNote X9 to collect all relevant studies from PubMed, Web of Science Core Collection, and PsycInfo. By the use of Covidence, the abstracts of potential studies were then independently screened by pairs of review authors, with disagreements resolved through discussion involving a third review author. Full-text publications of those studies deemed relevant by the abstract screening were also assessed similarly. Only studies adhering to the eligibility criteria presented in the PICO (Table 1) were included in the systematic review, and studies were assessed for quality before the best evidence synthesis was formed. Only high and medium quality studies were eligible for the evidence synthesis, while low quality studies were not sufficient to move forward to data extraction.

### Data Extraction

Systematic data extraction was employed for each included study to collect general characteristics such as first author, date of publication, geographical setting (country), study design, study population (sample size, age, industry), type and duration of intervention (see below), results, and quality assessment score.

Since we investigated the effectiveness of intervention on any outcomes related to work environment, safety and health the reporting of specific outcomes did not form part of our eligibility criteria. Thus, we did not exclude potential relevant studies due to heterogeneity in outcomes, as long as they represented a sound measure of these outcomes.

The outcomes were assessed and divided into eight overall outcome categories, and only one outcome measure within each category was included from each eligible study in the evidence synthesis. The outcome categories are (1) occupational injuries/accidents, (2) musculoskeletal disorders, (3) mental health problems, (4) ergonomic work factors, (5) psychosocial work factors, (6) safety, (7) Health and physical activity, and 8) physical capacity. The main outcomes for the present review were incidence of injury/accidents or other outcomes representing poor health such as musculoskeletal disorders, mental health problems, etc. (category 1–3 above). The outcomes employed from each included study were not limited to their primary outcome measure, but could also reflect secondary or tertiary outcome measures [26]. If several follow-up periods were reported, data from the longest follow-up period was employed for the evidence synthesis (unless specifically stated in the study aim that a given follow-up time was the primary focus of the study).

### Defining and Coding Included Interventions

Each included study was categorized by the intervention classification framework suggested by Dyreborg et.al [16]. This classification groups OHS interventions with the same type of mechanisms and theoretical foundations, which are expected to provide similar effects across settings, depending on the fidelity of the intervention and the contextual factors. We modified this framework (see the logic model in Fig. 1), which was primarily developed within safety research, to include ‘mental training’ as a new main category directed at the individual level, and ‘Virtual Reality Augmented attitude modifications’, as a new sub-category of ‘Attitude and Belief’-modification, also directed at the individual level. Thus, we categorized each study into nine main categories of interventions: (1) attitude and belief intervention; (2) behavior based intervention; (3) physiological modification, (4) mental training intervention; (5) culture and climate interventions; (6) organizational level interventions; (7) external interventions, such as legislation or enforcement; (8) multifaceted approaches (combining two or more main types of interventions): and (9) intervention type not included above. By using this framework, OSH interventions may be directed at the individual level, the group or organizational level, or across levels at the workplace. Furthermore, interventions may use one component or be multifaceted, and thus combine two or more main types of intervention components. Each of the included studies was coded by two or more researchers and the coding was discussed at project meetings until agreement was reached.

### Assessment of Quality

To assess the quality of each study, two authors (ES and KVGS) conducted an independent evaluation, and any discrepancies were discussed with the senior author (LLA) until a consensus was reached. For the methodological appraisal, we employed the quality assessment methods developed by the Institute for Work and Health (IWH), which comprised 16 unique questions (refer to Supplementary Material 2 for details). Each article was assigned an IWH quality assessment score based on a weighted sum score, with the weighting values of each question ranging from 1 to 3 [19, 20]. We calculated the rank score for each included study by dividing it by the maximal weighted sum score and multiplying it by 100. Finally, we categorized the studies into three groups based on their ranking score: low quality (below 50%), medium quality (50–85%), and high quality (> 85%) [19, 27]. We only included high and medium quality studies in the evidence synthesis [19, 28].

### Assessment of Level of Evidence

To clarify the evidence, we adopted the IWH-adapted “best evidence synthesis approach” (refer to Table 2), which considers the quality and quantity of articles evaluating the same intervention and finding consistency [19, 28]. We classified the level of evidence as ‘strong’, ‘moderate’, ‘limited’, ‘mixed’ or ‘insufficient’ based on the quality assessment of the included studies. A ‘strong’ level of evidence resulted in “recommendations” for practice, whereas a ‘moderate’ level of evidence resulted in “practice consideration” [19, 28]. However, an evidence level below moderate (i.e. limited, mixed or insufficient) led to the following message for practice: “Not enough evidence from the scientific literature to guide current policies/practices” [29]. It is important to note that this does not necessarily mean that the interventions may not be effective, but there is insufficient scientific evidence to extract conclusions [29].

**Table 2:** Best evidence synthesis guidelines (adapted from Kennedy et al. [19])

Level of evidence	Minimum quality	Minimum quantity	Consistency	Terminology for messages
Strong	High (> 85%)	Three	Three high quality studies agree If more than three studies, 3/4th of the medium and high quality studies agree	Recommendations
Moderate	Medium (50–85%)	Two high quality OR Two medium quality and one high quality	Two high quality studies agree OR Two medium quality studies and one high quality study agree. If more than three studies, more than 2/3rd of the medium and high quality studies agree	Practice considerations
Limited	Medium (50–85%)	One high quality OR Two medium quality OR One medium quality and one high quality	If two studies (medium and/or high quality), agree If more than two studies, more than 1/2 of the medium and high quality studies agree	
Mixed	Medium and high	Two	Findings from medium and high quality studies are contradictory	
Insufficient	No high quality studies, only one medium quality study, and/or any number of low quality studies			

To reach a ‘strong’ level of evidence, at least three high quality studies had to point in the same direction (i.e. all showing either positive, negative or no effect of the given intervention), or at least ¾ of all studies within a specific intervention category had to have the same direction of effect [29]. This is illustrated in the guidelines presented in Table 2.

We synthesized the level of evidence for high and medium quality studies for each specific intervention type within each of the 9 outcome domains agreed upon by the authors.

If deemed possible by the authors, due to both a practical and a data-driven approach, each outcome domain was further divided into relevant outcome categories. For instance, the domain of occupational injuries was divided into hand eczema. This allowed for a more specific and stakeholder-friendly evidence synthesis. When consensus was reached by the review team on the outcome domains and categories, evidence was synthesized for each domain and category.

## Results

### Study Selection

The search generated 4191 records from PsycInfo, 8316 from Web of Science and 13,569 from PubMed, in total of 26,076 records. After removing duplicates, 21,513 records were screened for title and abstract. After the screening, 21,257 records were excluded as they did not meet the eligibility criteria. After title and abstract screening, we assessed 256 articles from which 33 articles were included in our quality assessment and evidence synthesis (Fig. 2). Supplementary material 3 summarizes the study characteristics of the included studies.

### Data Extraction

All 33 studies were intervention studies, 20 studies were RCTs and 13 were non-RCTs. Study designs within the category of non-RCTs included: non-randomized controlled trials, randomized interventions, prospective controlled interventions, clinically controlled prospective interventions, quasi-experimental studies, and cross-over interventions.

11 included studies were published in the United States, four studies from Germany, three from Taiwan two from the UK, Iran, Australia and Denmark and one study from France, India, New Zealand, Romania, Switzerland, the Netherlands and Turkey. The majority of the studies were published within the last 10 years; only four studies were published before 2013. The majority of studies focused on young workers within health-related professions, such as nurses, junior doctors, surgeons, and anesthetists. However, several studies also examined interventions for apprentices in construction-related fields (such as masonry and metalwork), chefs, police officers and military recruits (see also Supplementary Material 3).

To see how the studies were distributed across age groups, we give a descriptive overview based on the following three age categories: < 18 years, 18–25 years and > 25 years. We used these age groups because more restrictive rules usually apply to young people under the age of 18. Three studies included workers < 18 years, 15 studies included workers 18–25 years and 8 studies included workers > 25 years. Two studies fell into two categories 18–25 and > 25 years and five studies did not report any age but insinuated young workers and were therefore included.

### Categorization Into Outcome Domains and Categories

The 33 studies were grouped into 9 outcome domains, mental health problems (*n* = 19), psychosocial work factors (*n* = 4), musculoskeletal disorders (*n* = 5), injuries (*n* = 3), hand eczema (*n* = 4), safety (*n* = 3), ergonomic work factors (n = 2), health and physical activity (*n* = 4) and physical capacity (*n* = 3). Studies with more than one outcome could be included in different domains.

### Quality Appraisal

Six of the included studies were classified as high quality (> 85% of criteria met) and 27 studies were classified as medium quality (50–85% of criteria met). No studies were classified as low quality (< 50% of criteria met).

### Evidence Synthesis

Level of evidence from the 33 high and medium quality studies was synthesized for each of the 9 outcome domains (outcomes measure) divided into types of OHS interventions. Where possible, we divided the outcome domains into subcategories. Level of evidence can be seen in Table 3.

**Table 3:** Level of evidence and accompanying messages for stakeholders

Outcome domain and intervention type	Studies	Consistency	Level of evidence	Message for stakeholders
**Mental health problems**	19			
1.1.0. Attitude and belief	10	6 effect (H = 3, M = 3) 4 no effect (H = 1, M = 3)	Limited (of a positive effect)	Not enough evidence from the scientific literature to guide current policies/practices
Stress	4	2 effect (H = 1, M = 1) 2 no effect (H = 0, M = 2)	Mixed	Not enough evidence from the scientific literature to guide current policies/practices
Anxiety	2	1 effect (H = 0, M = 1) 1 no effect (H = 0, M = 1)	Mixed	Not enough evidence from the scientific literature to guide current policies/practices
Burnout	2	0 effect (H = 0, M = 0) 2 no effect (H = 1, M = 1)	Limited (for no benefit)	Not enough evidence from the scientific literature to guide current policies/practices
1.2.0. Behavior based^*^	2	2 effect (H = 0, M = 2) 0 no effect (H = 0, M = 0)	Limited (of a positive effect)	Not enough evidence from the scientific literature to guide current policies/practices
1.4.0 Mental training	5	2 effect (H = 1, M = 1) 3 no effect (H = 1, M = 2)	Limited (for no benefit)	Not enough evidence from the scientific literature to guide current policies/practices
Stress	4	1 effect (H = 0, M = 1) 3 no effect (H = 1, M = 2)	Moderate (for no benefit)	**Practice consideration: Seek alternative interventions based on OHS experience/knowledge**
Anxiety	2	1 effect (H = 1, M = 0) 1 no effect (H = 0, M = 1)	Mixed	Not enough evidence from the scientific literature to guide current policies/practices
Burnout	2	0 effect (H = 0, M = 0) 2 no effect (H = 0, M = 2)	Limited (for no benefit)	Not enough evidence from the scientific literature to guide current policies/practices
2.1.0. Culture and climate	1	0 effect (H = 0, M = 0) 1 no effect (H = 0, M = 1)	Insufficient	Not enough evidence from the scientific literature to guide current policies/practices
4.0 Multifaceted	1	0 effect (H = 0, M = 0) 1 no effect (H = 0, M = 1)	Insufficient	Not enough evidence from the scientific literature to guide current policies/practices
**Psychosocial work factors**	4			
1.1.0. Attitude and belief	1	0 effect (H = 0, M = 0) 1 no effect (H = 0, M = 1)	Insufficient	Not enough evidence from the scientific literature to guide current policies/practices
1.2.0. Behavior based	1	1 effect (H = 0, M = 1) 0 no effect (H = 0, M = 0)	Insufficient	Not enough evidence from the scientific literature to guide current policies/practices
1.4.0 Mental training	1	0 effect (H = 0, M = 0) 1 no effect (H = 0, M = 1)	Insufficient	Not enough evidence from the scientific literature to guide current policies/practices
2.1.0. Culture and climate	1	1 effect (H = 0, M = 1) 0 no effect (H = 0, M = 0)	Insufficient	Not enough evidence from the scientific literature to guide current policies/practices
**Musculoskeletal disorders**	5			
1.3.0. Physiological modifications				
1.3.5. Support/compression	3	1 effect ( H = 0, M = 1) 2 no effect (H = 0, M = 2)	Limited (for no benefit)	Not enough evidence from the scientific literature to guide current policies/practices
1.3.1. Mixed physical training	1	0 effect ( H = 0, M = 0) 1 no effect (H = 0, M = 1)	Insufficient	Not enough evidence from the scientific literature to guide current policies/practices
4.0 Multifaceted	1	0 effect ( H = 0, M = 0) 1 no effect (H = 0, M = 1)	Insufficient	Not enough evidence from the scientific literature to guide current policies/practices
**Ergonomic work factors**	2			
4.0 Multifaceted	2	1 effect ( H = 0, M = 1) 1 no effect (H = 0, M = 1)	Mixed	Not enough evidence from the scientific literature to guide current policies/practices
**Injuries**	3			
1.3.0. Physiological modifications 1.3.5. Support/compression	2	1 effect ( H = 0, M = 1) 1 no effect (H = 0, M = 1)	Mixed	Not enough evidence from the scientific literature to guide current policies/practices
4.4 Multifaceted, across level	1	0 effect ( H = 0, M = 0) 1 no effect (H = 0, M = 1)	Insufficient	Not enough evidence from the scientific literature to guide current policies/practices
**Hand eczema**	4			
4.1 Multifaceted, individual level	4	3 effect ( H = 0, M = 3) 1 no effect (H = 0, M = 1)	Limited (of a positive effect)	Not enough evidence from the scientific literature to guide current policies/practices
**Safety**	3			
1.1.3 Teaching and education	1	1 effect ( H = 0, M = 1) 0 no effect (H = 0, M = 0)	Insufficient	Not enough evidence from the scientific literature to guide current policies/practices
4.1 Multifaceted, individual level	1	1 effect ( H = 0, M = 1) 0 no effect (H = 0, M = 0)	Insufficient	Not enough evidence from the scientific literature to guide current policies/practices
4.4 Multifaceted, across level	1	0 effect ( H = 0, M = 0) 1 no effect (H = 0, M = 1)	Insufficient	Not enough evidence from the scientific literature to guide current policies/practices
**Health and physical activity**	4			
1.1.0. Attitude and belief	1	0 effect ( H = 0, M = 0) 1 no effect (H = 1, M = 0)	Limited (for no benefit)	Not enough evidence from the scientific literature to guide current policies/practices
1.4.0 Mental training	1	1 effect ( H = 0, M = 1) 0 no effect (H = 0, M = 0)	Insufficient	Not enough evidence from the scientific literature to guide current policies/practices
4.0 Multifaceted	2	1 effect ( H = 0, M = 1) 1 no effect (H = 0, M = 1)	Mixed	Not enough evidence from the scientific literature to guide current policies/practices
**Physical capacity**	3			
1.3.0. Physiological modifications				
1.3.1. Mixed physical training	1	0 effect ( H = 0, M = 0) 1 no effect (H = 0, M = 1)	Insufficient	Not enough evidence from the scientific literature to guide current policies/practices
1.3.5. Support/compression	1	0 effect ( H = 0, M = 0) 1 no effect (H = 0, M = 1)	Insufficient	Not enough evidence from the scientific literature to guide current policies/practices
4.0 Multifaceted	1	0 effect ( H = 0, M = 0) 1 no effect (H = 0, M = 1)	Insufficient	Not enough evidence from the scientific literature to guide current policies/practices

*H* high quality, *M* medium quality

* = Both interventions reported on the outcome measure anxiety

#### Mental Health Problems

Nineteen studies were identified and grouped within the ‘mental health problems’ outcome domain [30–48]. Ten interventions were coded as ‘Attitude and belief’ [30–39], 2 interventions as ‘Behaviour based’ [40, 41], 5 interventions as ‘Mental training’ [42–46], 1 intervention as ‘Culture and climate’ [47] and 1 intervention ‘Multifaceted’ [48].

Of the 10 interventions coded as ‘Attitude and Belief’ [30–39], 6 showed a positive effect (3 high quality and 3 medium quality) [31–34, 37, 38] and 4 showed no benefits (1 high quality and 3 medium quality) [30, 35, 36, 39] on mental health problems leading to limited evidence of a positive effect and resulted in the following message for stakeholders: Not enough evidence from the scientific literature to guide current policies/practices.

The 2 interventions coded as ‘Behaviour based’ reported on anxiety [40, 41]. Both studies were of medium quality and showed a positive effect on anxiety leading to limited evidence of a positive effect and resulted in the following message for stakeholders: Not enough evidence from the scientific literature to guide current policies/practices.

Of the 5 interventions coded as ‘Mental training’ [42–46], 2 showed a positive effect (1 high quality and 1 medium quality) [43, 46] and 3 showed no benefits (1 high quality and 2 medium quality) [42, 44, 46] on mental health problems leading to limited evidence for no benefits and resulted in the following message for stakeholders: Not enough evidence from the scientific literature to guide current policies/practices.

When further dividing the interventions coded as ‘Mental training’ into specific outcomes for mental health problems, 1 intervention showed a positive effect on stress (medium quality) [43] and 3 interventions (1 high quality and 2 medium quality) showed no benefits on stress [42, 44, 45]. This resulted in moderate evidence for no benefits of mental training on stress and to the following message for stakeholders: ‘Seek alternative interventions based on OHS experience/knowledge’.

#### Psychosocial Work Factors

Four studies were identified and grouped within the ‘Psychosocial work factors’ outcome domain [37, 38, 45, 49]. One intervention was coded as ‘Attitude and Belief’ [38], 1 intervention as ‘Behaviour based’, 1 intervention as ‘Mental training’ [45] and 1 intervention as ‘Culture and climate’ [37]. Within this outcome domain, there was insufficient evidence for all intervention types and, therefore, not enough evidence from the scientific literature to guide current policies/practices.

#### Musculoskeletal Disorders

Five studies were identified and grouped within the ‘Musculoskeletal disorders’ outcome domain [50–54]. Three interventions were coded as ‘Physiological modifications—support/compression’, 1 intervention was coded as ‘Physiological modifications—mixed physical training’ and 1 intervention was coded as ‘Multifaceted’. Of the 3 interventions coded as ‘Physiological modifications—support/compression’, 1 intervention showed a positive effect (medium quality) [51] and 2 interventions showed no benefits (all of medium quality) on musculoskeletal disorders [50, 52] leading to limited evidence for no benefit and resulted in the following message for stakeholders: Not enough evidence from the scientific literature to guide current policies/practices.

#### Ergonomic Work Factors

Two studies were identified and grouped within the ‘Ergonomic work factors’ outcome domain, and both were coded as ‘Multifaceted’ [36, 54]. 1 intervention showed a positive effect (medium quality) [54] and 1 intervention showed no benefits (medium quality) [36] on ergonomic work factors leading to mixed evidence and resulted in the following message for stakeholders: Not enough evidence from the scientific literature to guide current policies/practices.

#### Injuries

Three studies were identified and grouped within the ‘Injuries’ outcome domain [25, 51, 52]. 1 intervention was coded as ‘Multifaceted across organizational levels’ [25] and 2 interventions as ‘Physiological modifications’ [51, 52]. We found mixed evidence of ‘Physiological modifications’ on injuries, resulting in the following message for stakeholders: Not enough evidence from the scientific literature to guide current policies/practices.

#### Hand Eczema

Four studies were identified and grouped within the ‘Hand eczema’ outcome domain and all were coded as’multifaceted individual level’ interventions [55–58]. 3 interventions showed a positive effect (all of medium quality) [55–57] and 1 intervention showed no benefits (medium quality) [58] on hand eczema leading to limited evidence of a positive effect and resulted in the following message for stakeholders: Not enough evidence from the scientific literature to guide current policies/practices.

#### Safety

3 studies were identified and grouped within the ‘Safety’ outcome domain and all three used a safety climate intervention approach [25, 54, 59]. 1 intervention was coded as ‘Teaching and education’ [59], 1 intervention was coded as’Multifaceted individual level’ [54] and 1 intervention was coded as’Multifaceted across organizational levels’ [25]. Within this outcome domain, there was insufficient evidence and, therefore, not enough evidence from the scientific literature to guide current policies/practices.

#### Health and Physical Activity

4 studies were identified and grouped within the ‘Health and physical activity’ outcome domain [30, 36, 43, 60]. 2 interventions were coded as ‘Multifaceted’ [36, 60], 1 intervention as ‘Mental training’ [43] and 1 intervention as ‘Attitude and belief’ [30]. 1 of the 2 ‘Multifaceted’ interventions showed a positive effect (medium quality) and the other showed no benefits (medium quality) leading to mixed evidence and resulted in the following message for stakeholders: Not enough evidence from the scientific literature to guide current policies/practices. The study within ‘Attitude and belief’ showed no benefits and was of high quality leading to limited evidence for no benefits.

#### Physical Capacity

Three studies were identified and grouped within the ‘Physical capacity’ outcome domain [36, 50, 61]. 1 intervention was coded as ‘Physiological modifications—mixed physical training’ [61] 1 intervention was coded as ‘Physiological modifications—support/compression’ [50] and 1 intervention was coded as ‘Multifaceted’ [36]. All studies were of medium quality showing no benefits and resulted in insufficient evidence and, therefore, not enough evidence from the scientific literature to guide current policies/practices.

## Discussion

The systematic review identified 33 suitable high or medium quality studies reporting on the effect of workplace interventions to support young workers’ safety, work environment and health.

The evidence synthesis revealed moderate evidence for no benefit of ‘Mental training’ interventions on stress. We found limited evidence of a positive effect of the following intervention types: ‘Attitude and belief’ on mental health problems, ‘Behavior based’ on anxiety, and ‘Multifaceted on an individual level’ on hand eczema. We found limited evidence for no benefit of the following intervention types: ‘Mental training’ on mental health problems, and ‘Physiological modifications—support/compression’ on musculoskeletal disorders. The remainder intervention types revealed mixed or insufficient evidence.

Overall, the evidence synthesis shows that there is not enough evidence from the scientific literature to guide current practices and emphasize the need for high quality intervention studies specifically aiming at increasing or maintaining young workers’ health, work environment and safety.

Importantly, the interventions included in the review focused mainly on individual measures such as ‘attitude and belief’, ‘mental training’, ‘teaching and education’, ‘physiological modifications’, and to a smaller extent organizational level measures and multifaceted interventions. There is thus a need for future studies that can investigate possible preventive measures at the group or organizational level, which are less dependent on the individual prerequisites of each young worker.

### Discussion of Study Results

In this review, we examined broad classifications of OHS interventions targeting young workers, as presented in Fig. 1. The various components of OHS interventions were used to classify the various types of approaches to improve OHS among young workers. Using this framework, OHS interventions may be directed at the individual level, the group or organizational level, or across levels at the workplace, and finally external approaches such as legislation and enforcement. Furthermore, interventions may use one component or be multifaceted, and thus combine two or more main types of intervention components. Multifaceted approaches may combine intervention components at the individual level or at the organizational level, or combine components across levels. The model of Dyreborg et al.[16] is a generalized model (logic model), that provides an understanding of the nature of safety interventions and possible pathways for prevention. Furthermore, this classification is useful to help classify safety approaches and avoid misclassifications. The goal was to provide an informed basis for stakeholders to select more effective approaches to reduce OHS problems among young workers. The process was framed in the conceptual model of Lund and Aarø [62], as presented in Fig. 1.

#### Mental Health Problems

We found moderate evidence for no benefit of ‘Mental training’ interventions on stress leading to the following message for stakeholders: Seek alternative interventions based on OHS experience/knowledge’. We found limited evidence of a positive effect of ‘Attitude and belief’ interventions on overall mental health problems and of ‘Behavior based’ interventions on anxiety. In addition, we found limited evidence of no benefit of ‘Mental training’ interventions on overall mental health problems.

Both Danish and international sources indicate that mental health symptoms constitute a special problem for the younger part of the population, and a previous study showed that young workers report more depression and anxiety symptoms than older workers [2]. However, various data suggest that the incidence of psychological symptoms and disorders is relatively high among children and young people already before working age. Although the workplace is, thus, assumed not to be the only cause of mental health symptoms that affect young people in the labour market, the workplace can, however, be a particularly relevant arena for prevention efforts aimed at this group. There can be good reasons for choosing to focus prevention efforts on young workers, and thus break a "vicious circle" in the sense of reducing the negative effect of mental health symptoms on productivity and work ability.

No previous reviews have focused on young workers and interventions to reduce mental health problems. However, a systematic meta-review identified empirically supported interventions that workplaces can utilize to aid in the prevention of common mental illness as well as facilitating the recovery of employees diagnosed with depression and/or anxiety [63]. They found moderate evidence for two primary prevention interventions; enhancing employee control and promoting physical activity. Stronger evidence was found for CBT-based stress management although less evidence was found for other secondary prevention interventions, such as counselling. Strong evidence was also found against the routine use of debriefing following trauma.

Our review revealed that more than half of the identified studies (19 out of 33) focused on mental health problems. The large number of studies in this field allowed for a further sub-categorization, revealing moderate evidence for no benefit of mental training on stress (constituting mindfulness or mindfulness in combination with meditation or yoga). This is somewhat surprising, as a recent scoping review of reviews demonstrated, that three workplace intervention types that have been largely researched (mindfulness, education and information provision and individual psychological therapies) all had positive effects on burnout/stress reduction [64]. Future studies should investigate whether younger workers may benefit more from organizational interventions focusing on e.g. healthy planning of work and safety climate rather than individually oriented interventions such as mindfulness and yoga for preventing mental health problems.

#### Psychosocial Work Factors

Four studies were identified and grouped within this outcome category, all reporting on different intervention types, leading to insufficient evidence.

A previous study showed that young workers report more depression and anxiety symptoms than older workers. However, the study also indicated, that young workers generally state that they have a better psychosocial work environment, except for influence at work, which was lower among young workers [2]. The latter finding may be a consequence of hierarchies related to seniority. A recent study on more than 300.000 young employees in Denmark found that several psychosocial work factors were associated with sickness absence spells of any length [11]. Specifically, they found that among women, employment in occupations with high quantitative demands, low decision authority, high job strain, high emotional demands, or high work-related physical violence was associated with higher rates of sickness absence.

This underscores the complexity of young workers’ experiences, suggesting that while they might perceive their psychosocial work environment as generally positive, factors such as high job demands, low decision authority, and exposure to emotional stress or physical violence contribute to their increased sickness absence, indicating a need for tailored interventions. A recent systematic review on the effectiveness of workplace interventions in improving psychosocial working conditions, health and retention of workers identified strong to moderate evidence on four different intervention approaches concerning two health-related outcomes [65]. More specifically, the review found that interventions focusing on “changes in work time arrangements”, “influence on work tasks and work organization”, “Health care approaches”, and more generally “improvements of the psychosocial work environment” were to be effective when deployed as an organizational level intervention. Moreover, the review found evidence of the efficacy of interventions on “burnout” and “various health and wellbeing outcomes” [65]. Accordingly, these experiences from interventions focusing on the general working population may also prove useful in inspiring intervention programs specifically targeting younger workers.

#### Musculoskeletal Disorders

We found limited evidence for no benefit of ‘Physiological modifications’ through the use of support and/or compression on musculoskeletal disorders.

The review highlights, that there are not many workplace interventions exclusively among young workers with a focus on treating or preventing musculoskeletal disorders. However, young people have taken part in many previous MSD interventions, as part of the total study population. There can be many reasons for this, but an important factor is undeniably time. It takes time before MSD occurs, therefore young people can be an overlooked group in primary prevention. MSD is therefore not as widespread among young people, which is supported by the lower proportion indicating that they have pain several times a week in this group compared to the middle-aged and the elderly. However, there is still a significant proportion who have pain, in addition to the excess of physical strain, which is why future research should focus more on early prevention among young workers.

A previous systematic review on the effectiveness of workplace interventions in rehabilitating musculoskeletal disorders found strong evidence for implementing strength training at the workplace to reduce MSD among workers with physically demanding employment. Although the included studies in the previous review did not provide age-stratified results, it is plausible that strength training at the workplace will also be effective for young people. By contrast, the previous review found that there was not enough evidence from the scientific literature to guide current practices regarding the effect of workplace ergonomic interventions [26]. Previous reviews on the general working population have both reported an effect, no effect and conflicting results of workplace ergonomics on MSD [26, 66–68].

#### Ergonomic Work Factors

Two studies were identified and grouped within this outcome category, both reporting on multifaceted interventions, leading to mixed evidence. This highlights, that there are very few workplace interventions exclusively among young workers with a focus on mitigating ergonomic risk factors at work. This is problematic, since (1) young workers report a higher amount of physically demanding work and (2) cumulative exposure to mechanical work factors (ergonomic work factors) may have adverse health effects over time for the young workers. This entails an increased risk of developing MSDs [69] and previous studies have shown an exposure–response association between ergonomic work factors such as lifting and kneeling and risk of long-term sickness absence [6, 7].

#### Injuries

Three studies were identified and grouped within this outcome category and they revealed mixed evidence of ‘Physiological modifications’ through the use of support and/or compression on injuries.

Young people’s increased risk of being injured as a result of accidents at work is well known and has been in focus for a long time. As mentioned in the introduction, young people’s safety at work has been part of several initiatives that have been launched, but as illustrated in this review, there is a lack of scientific research on interventions aiming at improving safety and reducing accidents/injuries specifically among young workers. When it comes to occupational accidents, the consequences are seen immediately in the form of acute occupational injuries (short latency) whereas, when it comes to other areas, such as physically demanding work and chemistry, we only see the consequences later in working life (long latency).

The 3 studies within this domain involved workplace injuries among construction apprentices, military trainees and army recruits. Thus, the few studies were heterogeneous in both types of injury and population.

#### Safety

Three studies were identified and grouped within the outcome category ‘safety’, all reporting on different types of interventions, leading to insufficient evidence.

An internationally acknowledged way of measuring safety at work is through the concept of "safety climate" and numerous studies today explicitly or implicitly regard safety climate as a leading indicator capable of predicting safety outcomes [70–75]. A study by Ajslev et al. found that a higher number of safety climate problems progressively increased the odds of reporting at least one accident within the last 12 months at the two-year follow-up [76].

The present review highlight, that there are very few studies that specifically examine safety measures among young people. This is in agreement with a previous review of young people’s working environment in the Nordics [3] where no studies were found describing the effect of interventions to prevent accidents among young people, but the report pointed out the need for studies in this area in the Nordic region.

Another review from the Nordic countries implies that interventions targeting organizational factors such as safety norms, safety culture, and climate may influence attitudes and behaviour and consequently also the injury risk of young workers [1]. A Campbell systematic review evaluated the relative effectiveness of workplace safety interventions, including all age groups, and found strong evidence that greater effects are achieved with safety interventions directed toward the group or organizational level rather than those directed at individuals, which in general, provided no effect or only very modest effect on the outcomes [16]. Multifaceted approaches that combined intervention elements on the organizational level or across levels provided moderate to strong effects, in particular when engineering controls were integrated with other components.

Even though the Campbell systematic review did not focus particularly on young workers, some studies on young workers were included, which is of interest to the present study. A study found no effect on cutting injuries among young and inexperienced workers following either safety training to improve safety in the use of case‐cutters or an engineering intervention [77]. Marlenga et al. [78] found no significant effect of external approaches (legislative efforts) on tractor events on roadways among farm youth. A third study assessed whether active dissemination and in-depth communication and counselling on the North American Guidelines for Children’s Agricultural Tasks reduced childhood agricultural injuries [79]. The intervention also included in‐depth communication between educators and intervention group participants and showed a significant effect on injuries.

#### Hand Eczema

Three of 4 studies showed a positive effect of ‘Multifaceted individual level’ interventions on hand eczema leading to limited evidence of a positive effect.

Occupational eczema often starts at a young age [80], and the eczema tends to become chronic [81]. It is therefore important to focus on preventive measures and to reduce exposure to skin irritants already from the beginning of entering the workforce. In a Swedish study, they found that young hairdressers had a higher incidence of hand eczema than controls [82], whereas, in a Danish study, 45% of the hairdressers gave hand eczema as a reason for their career change [83]. A review has previously suggested an association between occupational chemical substance exposure among young hairdressers and skin reactions, e.g., hand eczema [1] and found studies suggesting a possible effect of an educational skin protection program on skin reactions and hand eczema among young workers in the Nordic countries [55, 84].

The present review suggests limited evidence of a positive effect of a multifaceted intervention at the individual level of apprentice workers within hairdressing, health-related professions (e.g. nurses) and non-health professions (e.g. metal workers). The interventions were mainly characterized by education and training programs on skin protection and prevention of hand eczema. Even though 3 out of 4 studies pointed to a positive effect, the lack of a high quality study hampered the level of evidence generated in the evidence synthesis, resulting in a limited evidence level. Thus, there is a need for high quality studies investigating the effect of workplace interventions on hand eczema among young workers.

#### Health and Physical Activity

Four studies were identified and grouped within this outcome category. We found mixed level of evidence of ‘Multifaceted’ interventions and limited level of evidence of ‘Attitude and belief’ interventions on health and physical activity.

The interventional outcomes of the included studies consisted of sickness absence, physical activity and blood pressure. Thus, the outcomes within this domain, along with the intervention types (attitude and belief, multifaceted and mental training), were very heterogeneous.

#### Physical Capacity

The three identified studies focused on a physical conditioning program, lower-body compression garment and a social media multifaceted intervention, respectively. Targeted physical exercise at the workplace is well-documented to improve factors such as muscle strength and cardiovascular fitness. Further, a previous systematic review of workplace interventions found no benefits of back/lumbar support on musculoskeletal disorders [26].

### Strengths and Limitations

Including both RCTs and non-RCTs in the systematic review has advantages and limitations. While including non-RCTs may compromise the review’s internal validity and increase risk of bias in blinding and sequence generation, we used the IWH approach for quality assessment and best evidence synthesis to address this. The decision to include non-RCTs in the present reviews speaks to a broader understanding and comprehensive coverage of the topic. By contrast, relying solely on RCTs may restrict understanding of effective workplace interventions, where randomized and carefully controlled trials (RCTs) are not always feasible [26]. By including non-RCTs, we avoid excluding valuable information on interventions to support young workers’ safety, work environment and health. Our objective was to transparently present the best evidence for practitioners using best evidence synthesis and involving relevant stakeholders. To maximize practical relevance, we intentionally included non-RCTs as well. Among the 33 high and moderate quality studies included in the evidence synthesis, one-third (13 studies) were non-RCTs. This allowed us to incorporate valuable information that would have been excluded if only RCTs were considered. However, it is worth noting that we encountered a limited number of eligible intervention studies for inclusion in the review, underscoring the need for more research on work environment, safety, and health specifically tailored to this group of workers. Thus, when it comes to the effectiveness of workplace interventions to support young workers’ safety, work environment and health, only a small amount of scientific research of sufficient quality exists. This may be attributed to several factors. Firstly, research on young people often focuses on their education and transition to work, with less emphasis on their work environment, likely because the importance of education for youth employment is attracting a higher degree of attention from both policy and research. Additionally, specific efforts for young people in the workplace may be more difficult to perform, as they constitute only a smaller part of the total workforce at the workplace. Moreover, workplace improvements are often directed towards the overall workforce, with less attention given to the challenges of young workers as a distinct group.

Because we investigated the effectiveness of intervention on any outcomes related to work environment, safety and health, we did not exclude potentially relevant studies due to heterogeneity in outcomes, as long as they represented a sound measure of work environment, safety and health. As expected, substantial heterogeneity in the interventional outcome measures, intervention type, population of young workers (industry/profession), study designs, and workplace contexts did not allow for the conduction of a meta-analysis, which is also coherent with other reviews within the field of work-related interventions and health [20, 26, 29]. Instead, we employed the pre-planned best evidence synthesis approach developed by IWH, with the opportunity to provide practitioners with the requested evidence-based approach to better identify and implement more relevant and effective workplace solutions. However, this approach does not consider sample size since small study populations count to the same extent in the evidence synthesis as studies including a larger study sample. Due to the heterogeneity and lack of a high level of evidence for the effect of any of the workplace interventions, there was not enough evidence from the scientific literature to guide current practices for any of the specific workplace interventions except for a moderate level of evidence for no benefit of ‘Mental training’ on stress. Thus it was not possible to make specific recommendations for practitioners since the component, study population, and intervention type are so different, and it should be recognized that general conclusions about the effectiveness of workplace interventions for young workers should be made with care.

A strength of the study is actively involving relevant stakeholders throughout the review process, ensuring a high degree of practical relevance. By engaging stakeholders during the design and search strategy phases, a practical approach was adopted that enabled practitioners to contribute their knowledge, experience, and needs in suitable workplace solutions for young workers. It should however be mentioned, that the stakeholders represented workers employed in four different sectors/industries specifically relevant to young workers (i.e. ‘Retail and Trade’, ‘Restaurants and Bars’, ‘Building and Construction’, and ‘Residential institutions and Home Care’) and are therefore, not representative of all sectors/industries of workers in Denmark.

There is a need for a clear and appropriate definition of how different types of OHS interventions are classified, including the combination of components included in the intervention programs. Otherwise, there is a risk of misclassification of OHS interventions. We have used a classification of intervention components [16], based on safety intervention, and supplied this with some categories relevant to the present review. In this broad review, the classification scheme was useful in identifying and classifying different types of OHS interventions delivered across varied levels (individual, group, organization or external). The classification process allowed us to classify interventions uniformly, to minimize the risk of misclassifications. We acknowledge that the method could be improved, in particular including a better categorization of psychosocial interventions, including subcategories, in future studies. We should also be aware that developments in the field of occupational health and safety, including the introduction of new technologies such as the use of ‘Virtual Reality Augmented’ to achieve attitude change, will require such a classification to be updated regularly to see if the categories are still durable. However, for this broad class of OHS interventions aimed at young workers, we believe the classification provides a relevant and uniform coding of interventions.

The present review may be susceptible to publication bias. We aimed to include as many studies as possible, considering the limited availability of eligible studies on workplace interventions for young workers’ safety, work environment, and health. This may have led to a higher likelihood of including studies with positive results. Additionally, relying solely on English language studies could introduce bias by not representing the entirety of available evidence in the field of work environment and health. Therefore, the presence of a language restriction bias in this study cannot be disregarded.

Despite geographical imbalances, the inclusion of studies from 14 different countries adds a degree of international perspective and generalizability. Out of the 33 studies included in the review, 11 were from the USA, while 13 were from Europe, with only 2 coming from the Scandinavian countries. The majority of studies included in the review focused on young workers within health-related professions, such as nurses, junior doctors, surgeons, and anaesthetists. However, several studies also examined interventions for apprentices in construction-related fields (such as masonry and metalwork), chefs, police officers and military recruits (see also ‘Supplementary material 3’). Overall, it seems that the findings of the present study primarily reflect the effectiveness of interventions for young workers within health-related professions. Future longitudinal studies should focus on interventions aiming at improving health, work environment and safety among young workers in non-health-related professions.

Additionally, it is important to note that the studies included in the review focused on young workers with a mean age ranging from 15 to 29 years. As an employer, there are several matters that you must be aware of when you have employed young people under the age of 18 because more restrictive rules usually apply to young people under the age of 18 [1]. However, only three studies in the review involved young workers below the age of 18, and thus the findings may primarily pertain to young workers between the ages of 18 and 29.

## Conclusions

The systematic review identified 33 suitable high or medium quality studies reporting on the effect of workplace interventions to support young workers’ safety, work environment and health. The evidence synthesis showed a moderate level of evidence for no benefit of ‘Mental training’ on stress. We found limited evidence of a positive effect of the following intervention types: ‘Attitude and belief’ on mental health problems, ‘Behavior based’ on anxiety, and ‘Multifaceted’ on hand eczema. We found limited evidence for no benefit of the following intervention types: ‘Mental training’ on mental health problems, and ‘Physiological modifications’ on musculoskeletal disorders. The remaining intervention types showed mixed or insufficient evidence.

Except for a moderate level of evidence for no benefit of ‘Mental training’ on stress, the evidence synthesis recommends, that there is not enough evidence from the scientific literature to guide current practices. The results emphasizes a strong need for high quality interventions specifically aiming at increasing or maintaining young workers’ work environment, safety and health. Included studies focused mainly on individual measures, highlighting the need for studies investigating possible preventive measures at the group or organizational level.

## Supplementary Information

Below is the link to the electronic supplementary material.

Supplementary file1 (DOCX 37 KB)

Supplementary file2 (DOCX 14 KB)

Supplementary file3 (DOCX 141 KB)

## Data Availability

The data that support the findings of this review will be available from the corresponding author upon reasonable request.
