# Supplementary material for: Systematic Review of Workplace Interventions to Support Young Workers’ Safety, Work Environment and Health
Source: J Occup Rehabil. 2024 Apr 30;35(2):215–33. doi: 10.1007/s10926-024-10186-y (PMC12089187; doi:10.1007/s10926-024-10186-y)
Supplement: Supplementary file 1 — Supplementary file1 (DOCX 37 KB) [file 10926_2024_10186_MOESM1_ESM.docx]

Supplementary material 1: Search strategy

**Information sources**

| **Database** | **Search date** |
| --- | --- |
| PubMed incl. MEDLINE | 19/04-22 |
| Web of Science core collection | 19/04-22 |
| PsycINFO via OVID | 19/04-22 |

**Note**

- Search terms are adapted for each database
- Search strategy for each selected database is reported below

Search strategy:

PubMed incl. MEDLINE

2007 to 2022

| **PICO** | Search terms | Search string |
| --- | --- | --- |
| **Population** | Apprentice  Internship  Trainee  "young adult employ*  "young adult work*"  "working young adult"  "working young adults”  “adolescent employ*  “adolescent work*”  “working adolescent”  “working adolescents”  “young employ*”  “young work*”  “working young”  “youth employ*”  “youth work*”  “working youth”  "Adult employ*  "Adult work*"  "Working adult"  "Working adults”    "teen work*"  "working teens"  "working teenagers”  “teen employ*” | "teen work*"[Title/Abstract] OR "working teens"[Title/Abstract] OR "working teenagers"[Title/Abstract] OR "teen employ*"[Title/Abstract] OR ("apprentice"[Title/Abstract] OR "apprentices"[Title/Abstract] OR ("internship"[Title/Abstract] AND "residency"[Title/Abstract]) OR "internship"[Title/Abstract] OR "internships"[Title/Abstract] OR ("trainee"[Title/Abstract] OR "trainee s"[Title/Abstract] OR "trainees"[Title/Abstract]) OR ("young adult worker"[Title/Abstract] OR "young adult workers"[Title/Abstract]) OR "young adult workers"[Title/Abstract] OR "young adult employ*"[Title/Abstract] OR "working young adult"[Title/Abstract] OR "working young adults"[Title/Abstract] OR ("adolescent work"[Title/Abstract] OR "adolescent workers"[Title/Abstract]) OR ("working adolescent"[Title/Abstract] OR "adolescent employ*"[Title/Abstract] OR "working adolescents"[Title/Abstract]) OR "young employ*"[Title/Abstract] OR ("working young"[Title/Abstract] OR "youth employ*"[Title/Abstract] OR "youth work"[Title/Abstract] OR "youth worker"[Title/Abstract] OR "youth workers"[Title/Abstract] OR "working youth"[Title/Abstract]) OR ("young work"[Title/Abstract] OR "young worker"[Title/Abstract] OR "young workers"[Title/Abstract])) |
| **AND** |  |  |
| **Intervention** | "Absence management"  "Adequate supervision"  "Alternative work"  "Attendance management"  "Behavior therapy"  "Behaviour therapy"  "Behavioral intervention"  "Behavioral interventions"  "Behavioúral intervention"  "Behavioural interventions"  "Biofeedback training"  "Cognitive behavioural intervention"  "Cognitive behavioural interventions"  "Cognitive behavioral intervention"  "Cognitive behavioral interventions"  "Cognitive behavioral principles"  "Cognitive behavioural principles"  "Cognitive behavioral training"  "Cognitive behavioural training"  "Combined modality therapy"  "Compensation management"  "Complementary Therapies"  "Coordinated program"  "Counselling"  "Disability management"  "Disclosure management"  "Early intervention"  "Education"  "Employee assistance"  "Employer accommodation"  "Employer contact"  "Environmental intervention"  "Environmental interventions"  "Ergonomic approaches"  "Ergonomic intervention"  "Ergonomic interventions"  "Ergonomic program"  "Ergonomic programme"  "Ergonomics training"  "Ergonomics"  "Exercise"  "Exercise therapy"  "Exercise training"  “Flexible work"  "Functional abilities"  "Functional ability"  "Functional capacity"  "Functional training"  "Good practice"  "graded activity"  "Graded work"  "Guidelines as topic"  "Health care provider training"  "Health intervention"  "Health interventions”  "Health promotion"  "Healthcare provider training"  "Human resource training"  Intervention  "Job accommodation"  "Job person"  "Legislation enforcement"  "Light duties"  "Light duty"  "Light work"  "Modified duties"  "Modified duty"  "Modified job"  "Modified work"  "Motivational interviewing"  "Multidisciplinary intervention"  "Occupational health"  "Occupational health guidelines"  “Occupational health and safety”  “Occupational health and safety training”  “Occupational Injuries”  "Occupational intervention"  "Occupational management"  "Occupational rehabilitation"  "Occupational safety"  "Occupational Therapy"  “OHS interventions”  “OHS training”  "Organisational change"  "Organisational changes"  "Organizational change"  "Organizational changes"  “Occupational safety and health”  “Occupational safety and health training”  "OSH intervention"  "OSH interventions"  “OSH teaching”  “OSH training”  "Pain reduction"  "Participatory ergonomics"  "Physical Therapy Modalities"  "physical therapy"  "prevention and control”  "prevention strategies"  "Psychotherapy"  "Reasonable accommodation"  "Rest breaks"  “Rest break”  "Safety training"  "Secondary prevention"  "Self-management training"  "Self-management programme"  "Service coordination"  "Stress management"  "Structured rehabilitation program"  "Suitable duties"  "Supervisor"  "Supportive colleagues"  "Supportive manager"  "Technical change"  "Technical changes"  "Training"  "Vocational assessment"  "Vocational rehabilitation"  "Work adjustment"  "Work based"  "Work conditioning"  "Work disability management"  "Work hardening"  "Work intervention"  "Work interventions"  "Work modification"  "Work program"  "Work site"  "Work trial"  "Work trials"  "Work-directed interventions"  "Work-directed interventions"  "Working interventions"  "Working trials"  "Workplace intervention"  "Workplace interventions"  "Workplace linked"  "Workplace modification"  "Workplace"  "Workplace-based intervention"  “Workplace-based learning”  "Worksite visits"  "Worksite*"  "Workstation adjustment"  motivation  "Occupational Health Promotion"  psychosocial "stress prevention"  “cognitive behavioral therapy program”  “cognitive behavioural therapy program” | "Health promotion"[Title/Abstract] OR "Healthcare provider training"[Title/Abstract] OR "Human resource training"[Title/Abstract] OR "intervention"[Title/Abstract] OR "interventions"[Title/Abstract] OR "Job accommodation"[Title/Abstract] OR "Job person"[Title/Abstract] OR "Legislation enforcement"[Title/Abstract] OR "Light duties"[Title/Abstract] OR "Light duty"[Title/Abstract] OR "Light work"[Title/Abstract] OR "Modified duties"[Title/Abstract] OR "Modified duty"[Title/Abstract] OR "Modified job"[Title/Abstract] OR "Modified work"[Title/Abstract] OR "Motivational interviewing"[Title/Abstract] OR "Multidisciplinary intervention"[Title/Abstract] OR "Occupational health"[Title/Abstract] OR "Occupational health guidelines"[Title/Abstract] OR "Occupational health and safety"[Title/Abstract] OR "Occupational health and safety training"[Title/Abstract] OR "Occupational Injuries"[Title/Abstract] OR "Occupational intervention"[Title/Abstract] OR "Occupational management"[Title/Abstract] OR "Occupational rehabilitation"[Title/Abstract] OR "Occupational safety"[Title/Abstract] OR "Occupational Therapy"[Title/Abstract] OR "OHS interventions"[Title/Abstract] OR "OHS training"[Title/Abstract] OR "Organisational change"[Title/Abstract] OR "Organisational changes"[Title/Abstract] OR "Organizational change"[Title/Abstract] OR "Organizational changes"[Title/Abstract] OR "Occupational safety and health training"[Title/Abstract] OR "OSH intervention"[Title/Abstract] OR "OSH interventions"[Title/Abstract] OR "OSH teaching"[Title/Abstract] OR "OSH training"[Title/Abstract] OR "Pain reduction"[Title/Abstract] OR "Participatory ergonomics"[Title/Abstract] OR "Physical Therapy Modalities"[Title/Abstract] OR "physical therapy"[Title/Abstract] OR "prevention and control"[Title/Abstract] OR "prevention strategies"[Title/Abstract] OR "Psychotherapy"[Title/Abstract] OR "Reasonable accommodation"[Title/Abstract] OR "Rest breaks"[Title/Abstract] OR "Rest break"[Title/Abstract] OR "Safety training"[Title/Abstract] OR "Secondary prevention"[Title/Abstract] OR "Self-management training"[Title/Abstract] OR "Self-management programme"[Title/Abstract] OR "Service coordination"[Title/Abstract] OR "Stress management"[Title/Abstract] OR "Structured rehabilitation program"[Title/Abstract] OR "Suitable duties"[Title/Abstract] OR "Supervisor"[Title/Abstract] OR "Supportive colleagues"[Title/Abstract] OR "Supportive manager"[Title/Abstract] OR "Technical change"[Title/Abstract] OR "Technical changes"[Title/Abstract] OR "Training"[Title/Abstract] OR (("Education"[MeSH Subheading] OR "Education"[Title/Abstract] OR "Training"[Title/Abstract] OR "Education"[MeSH Terms] OR "train"[Title/Abstract] OR "train s"[Title/Abstract] OR "trained"[Title/Abstract] OR "training s"[Title/Abstract] OR "trainings"[Title/Abstract] OR "trains"[All Fields]) AND ("occup health saf"[Journal] OR ("occupational"[Title/Abstract] AND "health"[Title/Abstract] AND "and"[Title/Abstract] AND "safety"[All Fields]) OR "Occupational health and safety"[All Fields])) OR "Vocational assessment"[Title/Abstract] OR "Vocational rehabilitation"[Title/Abstract] OR "Work adjustment"[Title/Abstract] OR "Work based"[Title/Abstract] OR "Work conditioning"[Title/Abstract] OR "Work disability management"[Title/Abstract] OR "Work hardening"[Title/Abstract] OR "Work intervention"[Title/Abstract] OR "Work interventions"[Title/Abstract] OR "Work modification"[Title/Abstract] OR "Work program"[Title/Abstract] OR "Work site"[Title/Abstract] OR "Work trial"[Title/Abstract] OR "Work trials"[Title/Abstract] OR "Work-directed interventions"[Title/Abstract] OR "Working interventions"[Title/Abstract] OR "Working trials"[Title/Abstract] OR "Workplace intervention"[Title/Abstract] OR "Workplace interventions"[Title/Abstract] OR "Workplace linked"[Title/Abstract] OR "Workplace modification"[Title/Abstract] OR "Workplace"[Title/Abstract] OR "Workplace-based intervention"[Title/Abstract] OR "Workplace-based learning"[Title/Abstract] OR "Worksite visits"[Title/Abstract] OR "worksite*"[Title/Abstract] OR "Workstation adjustment"[Title/Abstract] OR ("Absence management"[Title/Abstract] OR "Adequate supervision"[Title/Abstract] OR "Alternative work"[Title/Abstract] OR "Attendance management"[Title/Abstract] OR "Behavior therapy"[Title/Abstract] OR "Behaviour therapy"[Title/Abstract] OR "Behavioral intervention"[Title/Abstract] OR "Behavioral interventions"[Title/Abstract] OR "Behavioural intervention"[Title/Abstract] OR "Behavioural interventions"[Title/Abstract] OR "Biofeedback training"[Title/Abstract] OR "Cognitive behavioural intervention"[Title/Abstract] OR "Cognitive behavioural interventions"[Title/Abstract] OR "Cognitive behavioral intervention"[Title/Abstract] OR "Cognitive behavioral interventions"[Title/Abstract] OR "Cognitive behavioral principles"[Title/Abstract] OR "Cognitive behavioural principles"[Title/Abstract] OR "Cognitive behavioral training"[Title/Abstract] OR "Cognitive behavioural training"[Title/Abstract] OR "Combined modality therapy"[Title/Abstract] OR "Compensation management"[Title/Abstract] OR "Complementary Therapies"[Title/Abstract] OR "Coordinated program"[Title/Abstract] OR "Counselling"[Title/Abstract] OR "Disability management"[Title/Abstract] OR "Disclosure management"[Title/Abstract] OR "Early intervention"[Title/Abstract] OR "Education"[Title/Abstract] OR "Employee assistance"[Title/Abstract] OR "Employer accommodation"[Title/Abstract] OR "Employer contact"[Title/Abstract] OR "Environmental intervention"[Title/Abstract] OR "Environmental interventions"[Title/Abstract] OR "Ergonomic approaches"[Title/Abstract] OR "Ergonomic intervention"[Title/Abstract] OR "Ergonomic interventions"[Title/Abstract] OR "Ergonomic program"[Title/Abstract] OR "Ergonomic programme"[Title/Abstract] OR "Ergonomics training"[Title/Abstract] OR "Ergonomics"[Title/Abstract] OR "Exercise"[Title/Abstract] OR "Exercise therapy"[Title/Abstract] OR "Exercise training"[Title/Abstract] OR "Flexible work"[Title/Abstract] OR "Functional abilities"[Title/Abstract] OR "Functional ability"[Title/Abstract] OR "Functional capacity"[Title/Abstract] OR "Functional training"[Title/Abstract] OR "Good practice"[Title/Abstract] OR "graded activity"[Title/Abstract] OR "Graded work"[Title/Abstract] OR "Guidelines as topic"[Title/Abstract] OR "Health care provider training"[Title/Abstract] OR "Health intervention"[Title/Abstract] OR "Health interventions"[All Fields]) OR ("motivate"[Title/Abstract] OR "motivated"[Title/Abstract] OR "motivates"[Title/Abstract] OR "motivating"[Title/Abstract] OR "motivation"[MeSH Terms] OR "motivation"[Title/Abstract] OR "motivations"[Title/Abstract] OR "motive"[Title/Abstract] OR "motivational"[Title/Abstract] OR "motivator"[Title/Abstract] OR "motivators"[Title/Abstract] OR "motives"[Title/Abstract] OR "Occupational Health Promotion"[Title/Abstract] OR ("psychosocial"[Title/Abstract] OR "psychosocially"[All Fields]) OR "stress prevention"[All Fields]) OR "cognitive behavioral therapy program"[Title/Abstract] OR "cognitive behavioural therapy program"[Title/Abstract] |
| **AND** |  |  |
| **Not filter review** |  | ((((((((letter [pt] OR newspaper article [pt]))) OR (((systematic review [ti] OR meta-analysis [pt] OR meta-analysis [ti] OR systematic literature review [ti] OR this systematic review [tw] OR pooling project [tw] OR (systematic review [tiab] AND review [pt]) OR meta synthesis [ti] OR meta-analy*[ti] OR integrative review [tw] OR integrative research review [tw] OR rapid review [tw] OR umbrella review [tw] OR consensus development conference [pt] OR practice guideline [pt] OR drug class reviews [ti] OR cochrane database syst rev [ta] OR acp journal club [ta] OR health technol assess [ta] OR evid rep technol assess summ [ta] OR jbi database system rev implement rep [ta])))) OR review [pt]) OR ((review [tiab] OR reviews [tiab]))) OR meta-analy* [tw]) OR bibliography [tiab]) OR bibliographies [tiab] |
| **Not Intervention Mesh terms** |  | ("Methods"[MeSH Terms] OR "Methods"[MeSH Subheading] OR "Percutaneous Coronary Intervention"[MeSH Terms] OR "Early Medical Intervention"[MeSH Terms] OR "Genetic Engineering"[MeSH Terms]) |
| **Combined search string** |  | ((("teen work*"[Title/Abstract] OR "working teens"[Title/Abstract] OR "working teenagers"[Title/Abstract] OR "teen employ*"[Title/Abstract] OR ("apprentice"[Title/Abstract] OR "apprentices"[Title/Abstract] OR ("internship"[Title/Abstract] AND "residency"[Title/Abstract]) OR "internship"[Title/Abstract] OR "internships"[Title/Abstract] OR ("trainee"[Title/Abstract] OR "trainee s"[Title/Abstract] OR "trainees"[Title/Abstract]) OR ("young adult worker"[Title/Abstract] OR "young adult workers"[Title/Abstract]) OR "young adult workers"[Title/Abstract] OR "young adult employ*"[Title/Abstract] OR "working young adult"[Title/Abstract] OR "working young adults"[Title/Abstract] OR ("adolescent work"[Title/Abstract] OR "adolescent workers"[Title/Abstract]) OR ("working adolescent"[Title/Abstract] OR "adolescent employ*"[Title/Abstract] OR "working adolescents"[Title/Abstract]) OR "young employ*"[Title/Abstract] OR ("working young"[Title/Abstract] OR "youth employ*"[Title/Abstract] OR "youth work"[Title/Abstract] OR "youth worker"[Title/Abstract] OR "youth workers"[Title/Abstract] OR "working youth"[Title/Abstract]) OR ("young work"[Title/Abstract] OR "young worker"[Title/Abstract] OR "young workers"[Title/Abstract]))) AND ("Health promotion"[Title/Abstract] OR "Healthcare provider training"[Title/Abstract] OR "Human resource training"[Title/Abstract] OR "intervention"[Title/Abstract] OR "interventions"[Title/Abstract] OR "Job accommodation"[Title/Abstract] OR "Job person"[Title/Abstract] OR "Legislation enforcement"[Title/Abstract] OR "Light duties"[Title/Abstract] OR "Light duty"[Title/Abstract] OR "Light work"[Title/Abstract] OR "Modified duties"[Title/Abstract] OR "Modified duty"[Title/Abstract] OR "Modified job"[Title/Abstract] OR "Modified work"[Title/Abstract] OR "Motivational interviewing"[Title/Abstract] OR "Multidisciplinary intervention"[Title/Abstract] OR "Occupational health"[Title/Abstract] OR "Occupational health guidelines"[Title/Abstract] OR "Occupational health and safety"[Title/Abstract] OR "Occupational health and safety training"[Title/Abstract] OR "Occupational Injuries"[Title/Abstract] OR "Occupational intervention"[Title/Abstract] OR "Occupational management"[Title/Abstract] OR "Occupational rehabilitation"[Title/Abstract] OR "Occupational safety"[Title/Abstract] OR "Occupational Therapy"[Title/Abstract] OR "OHS interventions"[Title/Abstract] OR "OHS training"[Title/Abstract] OR "Organisational change"[Title/Abstract] OR "Organisational changes"[Title/Abstract] OR "Organizational change"[Title/Abstract] OR "Organizational changes"[Title/Abstract] OR "Occupational safety and health training"[Title/Abstract] OR "OSH intervention"[Title/Abstract] OR "OSH interventions"[Title/Abstract] OR "OSH teaching"[Title/Abstract] OR "OSH training"[Title/Abstract] OR "Pain reduction"[Title/Abstract] OR "Participatory ergonomics"[Title/Abstract] OR "Physical Therapy Modalities"[Title/Abstract] OR "physical therapy"[Title/Abstract] OR "prevention and control"[Title/Abstract] OR "prevention strategies"[Title/Abstract] OR "Psychotherapy"[Title/Abstract] OR "Reasonable accommodation"[Title/Abstract] OR "Rest breaks"[Title/Abstract] OR "Rest break"[Title/Abstract] OR "Safety training"[Title/Abstract] OR "Secondary prevention"[Title/Abstract] OR "Self-management training"[Title/Abstract] OR "Self-management programme"[Title/Abstract] OR "Service coordination"[Title/Abstract] OR "Stress management"[Title/Abstract] OR "Structured rehabilitation program"[Title/Abstract] OR "Suitable duties"[Title/Abstract] OR "Supervisor"[Title/Abstract] OR "Supportive colleagues"[Title/Abstract] OR "Supportive manager"[Title/Abstract] OR "Technical change"[Title/Abstract] OR "Technical changes"[Title/Abstract] OR "Training"[Title/Abstract] OR (("Education"[MeSH Subheading] OR "Education"[Title/Abstract] OR "Training"[Title/Abstract] OR "Education"[MeSH Terms] OR "train"[Title/Abstract] OR "train s"[Title/Abstract] OR "trained"[Title/Abstract] OR "training s"[Title/Abstract] OR "trainings"[Title/Abstract] OR "trains"[All Fields]) AND ("occup health saf"[Journal] OR ("occupational"[Title/Abstract] AND "health"[Title/Abstract] AND "and"[Title/Abstract] AND "safety"[All Fields]) OR "Occupational health and safety"[All Fields])) OR "Vocational assessment"[Title/Abstract] OR "Vocational rehabilitation"[Title/Abstract] OR "Work adjustment"[Title/Abstract] OR "Work based"[Title/Abstract] OR "Work conditioning"[Title/Abstract] OR "Work disability management"[Title/Abstract] OR "Work hardening"[Title/Abstract] OR "Work intervention"[Title/Abstract] OR "Work interventions"[Title/Abstract] OR "Work modification"[Title/Abstract] OR "Work program"[Title/Abstract] OR "Work site"[Title/Abstract] OR "Work trial"[Title/Abstract] OR "Work trials"[Title/Abstract] OR "Work-directed interventions"[Title/Abstract] OR "Working interventions"[Title/Abstract] OR "Working trials"[Title/Abstract] OR "Workplace intervention"[Title/Abstract] OR "Workplace interventions"[Title/Abstract] OR "Workplace linked"[Title/Abstract] OR "Workplace modification"[Title/Abstract] OR "Workplace"[Title/Abstract] OR "Workplace-based intervention"[Title/Abstract] OR "Workplace-based learning"[Title/Abstract] OR "Worksite visits"[Title/Abstract] OR "worksite*"[Title/Abstract] OR "Workstation adjustment"[Title/Abstract] OR ("Absence management"[Title/Abstract] OR "Adequate supervision"[Title/Abstract] OR "Alternative work"[Title/Abstract] OR "Attendance management"[Title/Abstract] OR "Behavior therapy"[Title/Abstract] OR "Behaviour therapy"[Title/Abstract] OR "Behavioral intervention"[Title/Abstract] OR "Behavioral interventions"[Title/Abstract] OR "Behavioural intervention"[Title/Abstract] OR "Behavioural interventions"[Title/Abstract] OR "Biofeedback training"[Title/Abstract] OR "Cognitive behavioural intervention"[Title/Abstract] OR "Cognitive behavioural interventions"[Title/Abstract] OR "Cognitive behavioral intervention"[Title/Abstract] OR "Cognitive behavioral interventions"[Title/Abstract] OR "Cognitive behavioral principles"[Title/Abstract] OR "Cognitive behavioural principles"[Title/Abstract] OR "Cognitive behavioral training"[Title/Abstract] OR "Cognitive behavioural training"[Title/Abstract] OR "Combined modality therapy"[Title/Abstract] OR "Compensation management"[Title/Abstract] OR "Complementary Therapies"[Title/Abstract] OR "Coordinated program"[Title/Abstract] OR "Counselling"[Title/Abstract] OR "Disability management"[Title/Abstract] OR "Disclosure management"[Title/Abstract] OR "Early intervention"[Title/Abstract] OR "Education"[Title/Abstract] OR "Employee assistance"[Title/Abstract] OR "Employer accommodation"[Title/Abstract] OR "Employer contact"[Title/Abstract] OR "Environmental intervention"[Title/Abstract] OR "Environmental interventions"[Title/Abstract] OR "Ergonomic approaches"[Title/Abstract] OR "Ergonomic intervention"[Title/Abstract] OR "Ergonomic interventions"[Title/Abstract] OR "Ergonomic program"[Title/Abstract] OR "Ergonomic programme"[Title/Abstract] OR "Ergonomics training"[Title/Abstract] OR "Ergonomics"[Title/Abstract] OR "Exercise"[Title/Abstract] OR "Exercise therapy"[Title/Abstract] OR "Exercise training"[Title/Abstract] OR "Flexible work"[Title/Abstract] OR "Functional abilities"[Title/Abstract] OR "Functional ability"[Title/Abstract] OR "Functional capacity"[Title/Abstract] OR "Functional training"[Title/Abstract] OR "Good practice"[Title/Abstract] OR "graded activity"[Title/Abstract] OR "Graded work"[Title/Abstract] OR "Guidelines as topic"[Title/Abstract] OR "Health care provider training"[Title/Abstract] OR "Health intervention"[Title/Abstract] OR "Health interventions"[All Fields]) OR ("motivate"[Title/Abstract] OR "motivated"[Title/Abstract] OR "motivates"[Title/Abstract] OR "motivating"[Title/Abstract] OR "motivation"[MeSH Terms] OR "motivation"[Title/Abstract] OR "motivations"[Title/Abstract] OR "motive"[Title/Abstract] OR "motivational"[Title/Abstract] OR "motivator"[Title/Abstract] OR "motivators"[Title/Abstract] OR "motives"[Title/Abstract] OR "Occupational Health Promotion"[Title/Abstract] OR ("psychosocial"[Title/Abstract] OR "psychosocially"[All Fields]) OR "stress prevention"[All Fields]) OR "cognitive behavioral therapy program"[Title/Abstract] OR "cognitive behavioural therapy program"[Title/Abstract])) NOT ("letter"[Publication Type] OR "newspaper article"[Publication Type] OR ("systematic review"[Title] OR "meta-analysis"[Publication Type] OR "meta-analysis"[Title] OR "systematic literature review"[Title] OR "this systematic review"[Text Word] OR "pooling project"[Text Word] OR ("systematic review"[Title/Abstract] AND "review"[Publication Type]) OR "meta synthesis"[Title] OR "meta analy*"[Title] OR "integrative review"[Text Word] OR "integrative research review"[Text Word] OR "rapid review"[Text Word] OR "umbrella review"[Text Word] OR "consensus development conference"[Publication Type] OR "practice guideline"[Publication Type] OR "drug class reviews"[Title] OR "cochrane database syst rev"[Journal] OR "acp j club"[Journal] OR "health technol assess"[Journal] OR "evid rep technol assess summ"[Journal] OR "jbi database system rev implement rep"[Journal]) OR "review"[Publication Type] OR ("review"[Title/Abstract] OR "reviews"[Title/Abstract]) OR "meta analy*"[Text Word] OR "bibliography"[Title/Abstract] OR "bibliographies"[Title/Abstract])) NOT ("Methods"[MeSH Terms] OR "Methods"[MeSH Subheading] OR "Percutaneous Coronary Intervention"[MeSH Terms] OR "Early Medical Intervention"[MeSH Terms] OR "Genetic  Engineering"[MeSH Terms]) |
| **filter** |  | Year and language |

## Web of science core collection

2007 to 2022

| **PICO** | Search terms | Search string |
| --- | --- | --- |
| **Population** | Apprentice  Internship  Trainee  "young adult employ*”  "young adult work*"  "working young adult"  “adolescent employ*”  “adolescent work*”  “working adolescent”  “young employ*”  “young work*”  “working young”  “youth employ*”  “youth work*”  “working youth”  "teen work*"  “teenager work*”  "working teen"  "working teenager”  “teen employ*”  “teenager employ*” | TI=("young adult employ*”) OR AB=("young adult employ*”) OR TI=("young adult work*") OR AB=("young adult work*") OR TI=("working young adult") OR AB=("working young adult") OR TI=(“adolescent employ*") OR AB=(“adolescent employ*") OR TI=(“adolescent work*”) OR AB=(“adolescent work*”) OR TI=(“working adolescent”) OR AB=(“working adolescent”) OR TI=(“young employ*”) OR AB=(“young employ*”) OR TI=(“young work*”) OR AB=(“young work*”) OR TI=(“working young”) OR AB=(“working young”) OR TI=(“youth employ*”) OR AB=(“youth employ*”) OR TI=(“youth work*”) OR AB=(“youth work*”) OR TI=(“working youth”) OR AB=(“working youth”) OR TI=("teen work*") OR AB=("teen work*") OR TI=(“teenager work*”) OR AB=(“teenager work*”) OR TI=("working teen") OR AB=("working teen") OR TI=("working teenager”) OR AB=("working teenager”) OR TI=(“teen employ*”) OR AB=(“teen employ*”) OR TI=(“teenager employ*”) OR AB=(“teenager employ*”) OR TI=(Trainee) OR AB=(Trainee) OR TI=(Internship) OR AB=(Internship) OR AB=(Apprentice) OR TI=(Apprentice) |
| **AND** |  |  |
| **Intervention** | "Absence management"  "Adequate supervision"  "Alternative work"  "Attendance management"  "Behavi* therapy"  "Cognitive behavi* principles"  "Combined modality therap*"  "Compensation management"  "Complementary Therap*"  "Coordinated program"  Counselling  "Disability management"  "Disclosure management"  Education  "Employ* assistance"  "Employ* accommodation"  "Employ* contact"  Ergonomic*  Exercise*  “Flexible work"  "Functional ability*"  "Functional capacit*"  "Good practice*"  "graded activit*"  "Graded work"  "Health promotion*"  Intervention  "Job accommodation*"  "Job person"  "Legislation enforcement"  "Light dut*"  "Light work"  "Modified dut*"  "Modified job"  "Modified work"  "Motivational interviewing"  "Occupational health"  “Occupational Injur*”  "Occupational management"  "Occupational rehabilitation"  "Occupational safety"  "Occupational Therap*"  “OHS learning”  “OHS teaching”  "Organisational change"  "Organizational change"  “Occupational safety and health”  “OSH learning”  “OSH teaching”  "Pain reduction"  "physical therap*"  "prevention and control”  "prevention strateg*"  "Psychotherapy"  "Reasonable accommodation"  "Reasons adjustment"  “Rest break*”  "Self-management program*"  "Service coordination"  "Stress management"  "Structured rehabilitation program*"  "Suitable dut*"  "Supervisor"  "Supportive colleagues"  "Supportive manager"  "Technical change"  Training  "Vocational assessment"  "Vocational rehabilitation"  "Work adjustment"  "Work based"  "Work conditioning"  "Work disability management"  "Work hardening"  "Work modification"  "Work program"  "Work site"  Workplace  Worksite*  "Workstation adjustment"  motivation  "Occupational Health Promotion"  "stress prevention"  “cognitive behavioral therapy program” | ((((((((((((((((((((((((((((((((((((((((((((((((((((((((((((((((((((((((((((((((((((((((TS=("Absence management")) OR TS=("Adequate supervision" )) OR TS=("Alternative work")) OR TS=("Attendance management")) OR TS=("Behavi* therapy")) OR TS=("Cognitive behavi* principles")) OR TS=("Combined modality therap*")) OR TS=("Compensation management")) OR TS=("Complementary Therap*")) OR TS=("Coordinated program")) OR TS=(Counselling)) OR TS=("Disability management")) OR TS=("Disclosure management")) OR TI=(Education)) OR AB=(Education)) OR TS=("Employ* assistance")) OR TS=("Employ* accommodation")) OR TS=("Employ* contact")) OR TS=(Ergonomic*)) OR TI=(Exercise*)) OR AB=(Exercise*)) OR TS=(“Flexible work")) OR TS=("Functional abilit*" )) OR TS=("Functional capacit*")) OR TS=("Good practice*")) OR TS=("graded activit*")) OR TS=("Graded work")) OR TS=("Health promotion*" )) OR TI=(Intervention)) OR AB=(Intervention)) OR TS=("Job accommodation*")) OR TS=("Job person" )) OR TS=("Legislation enforcement" )) OR TS=("Light dut*")) OR TS=("Light work" )) OR TS=("Modified dut*")) OR TS=("Modified job")) OR TS=("Modified work")) OR TS=("Motivational interviewing" )) OR TS=("Occupational health")) OR TS=(“Occupational Injur*”)) OR TS=("Occupational management")) OR TS=("Occupational rehabilitation" )) OR TS=("Occupational safety")) OR TS=("Occupational Therap*")) OR TS=(“OHS learning”)) OR TS=(“OHS teaching”)) OR TS=("Organisational change" )) OR TS=("Organizational change" )) OR TS=(“Occupational safety and health”)) OR TS=(“OSH learning”)) OR TS=(“OSH teaching”)) OR TS=("Pain reduction")) OR TS=("Physical Therap*")) OR TS=("prevention and control”)) OR TS=("prevention strateg*")) OR TS=("Psychotherapy" )) OR TS=("Reasonable accommodation")) OR TS=("Reasons adjustment")) OR TS=(“Rest break*”)) OR TS=("Self-management program*")) OR TS=("Service coordination" )) OR TS=("Stress management")) OR TS=("Structured rehabilitation program*")) OR TS=("Suitable dut*")) OR TS=("Supervisor")) OR TS=("Supportive colleagues")) OR TS=("Supportive manager")) OR TS=("Technical change" )) OR TI=(Training)) OR AB=(Training)) OR TS=("Vocational assessment")) OR TS=("Vocational rehabilitation")) OR TS=("Work adjustment")) OR TS=("Work based" )) OR TS=("Work conditioning")) OR TS=("Work disability management")) OR TS=("Work hardening")) OR TS=("Work modification")) OR TS=("Work program")) OR TS=("Work site" )) OR TS=("Work trial*")) OR TS=(Workplace)) OR TS=(Worksite* )) OR TS=("Workstation adjustment")) OR TS=(motivation)) OR TS=("Occupational Health Promotion")) OR TS=("stress prevention")) OR TS=(“cognitive behavioral therapy program”) |
| **AND** |  |  |
| **Filters “web of science categories”** |  |  |

## PsycINFO via OVID

2007 to 2022

| **PICO** | Search term | Search string |
| --- | --- | --- |
| **Population** | Apprentice  Internship  Trainee  young adult employ*  young adult work*  adolescent employ*  adolescent work*  working adolescent  young employ*  young work*  working young  youth employ*  youth work*  working youth  teen work*  teenager work*  working teen  teen employ* | internship.ab,ti. Apprentice.ab,ti. trainee.ab,ti.  young adult employ*.mp. young adult work*.mp. adolescent employ*.mp. adolescent work*.mp. working adolescent.mp. young employ*.mp. young work*.mp. working young.mp. youth employ*.mp. youth work*.mp. working youth.mp. teen work*.mp. teenager work*.mp. working teen.mp. working teenager.mp. teen employ*.mp. |
| **AND** |  |  |
| **Intervention** | Absence management  Adequate supervision  Alternative work  Attendance management  Behavi* therapy  Cognitive behavi* principles  Combined modality therap*  Compensation management  Complementary Therap*  Coordinated program  Counselling  Disability management  Disclosure management  Education  Employ* assistance  Employ* accommodation  Employ* contact  Ergonomic*  Exercise*  Flexible work  Functional ability*  Functional capacit*  Good practice*  graded activit*  Graded work  Health promotion*  Intervention  Job accommodation*  Job person  Legislation enforcement  Light dut*  Light work  Modified dut*  Modified job  Modified work  Motivational interviewing  Occupational health  Occupational Injur*  Occupational management  Occupational rehabilitation  Occupational safety  Occupational Therap*  Organisational change  Organizational change  Occupational safety and health  Pain reduction  physical therap*  prevention and control  prevention strateg*  Psychotherapy  Reasonable accommodation  Reasons adjustment  Rest break*  Self-management program*  Service coordination  Stress management  Structured rehabilitation program*  Suitable dut*  Supervisor  Supportive colleagues  Supportive manager  Technical change  Training  Vocational assessment  Vocational rehabilitation  Work adjustment  Work based  Work conditioning  Work disability management  Work hardening  Work modification  Work program  Work site  Work trial*  Workplace  Worksite*  Workstation adjustment  motivation  Occupational Health Promotion  stress prevention  cognitive behavioral therapy program | Absence management.ab,ti.  Adequate supervision.ab,ti.  Alternative work.ab,ti.  Attendance management.ab,ti.  "Behavi* therapy".ab,ti.  "Cognitive behavi* principles".ab,ti.  "Combined modality therap*".ab,ti.  Compensation management.ab,ti.  "Complementary Therap*".ab,ti.  Coordinated program.ab,ti.  Counselling.ab,ti.  Disability management.ab,ti.  Disclosure management.ab,ti.  Education.ab,ti.  "Employ* assistance".ab,ti.  "Employ* accommodation".ab,ti.  "Employ* contact".ab,ti.  "Ergonomic*".ab,ti.  "Exercise*".ab,ti.  Flexible work.ab,ti.  "Functional ability*".ab,ti.  "Functional capacit*".ab,ti.  "Good practice*".ab,ti.  "graded activit*".ab,ti.  Graded work.ab,ti.  "Health promotion*".ab,ti.  Intervention.ab,ti.  "Job accommodation*".ab,ti.  Job person.ab,ti.  Legislation enforcement.ab,ti.  "Light dut*".ab,ti.  Light work.ab,ti.  "Modified dut*".ab,ti.  Modified job.ab,ti.  Modified work.ab,ti.  Motivational interviewing.ab,ti.  Occupational health.ab,ti.  "Occupational Injur*".ab,ti.  Occupational management.ab,ti.  Occupational rehabilitation.ab,ti.  Occupational safety.ab,ti.  "Occupational Therap*".ab,ti.  OHS learning.ab,ti.  OHS teaching.ab,ti.  Organisational change.ab,ti.  Organizational change.ab,ti.  (Occupational safety and health).ab,ti.  OSH learning.ab,ti.  OSH teaching.ab,ti.  Pain reduction.ab,ti.  "physical therap*".ab,ti.  (prevention and control).ab,ti.  "prevention strateg*".ab,ti.  Psychotherapy.ab,ti.  Reasonable accommodation.ab,ti.  Reasons adjustment.ab,ti.  "Rest break*".ab,ti.  "Self-management program*".ab,ti.  Service coordination.ab,ti.  Stress management.ab,ti.  "Structured rehabilitation program*".ab,ti.  "Suitable dut*".ab,ti.  Supervisor.ab,ti.  Supportive colleagues.ab,ti.  Supportive manager.ab,ti.  Technical change.ab,ti.  Training.ab,ti.  Vocational assessment.ab,ti.  Vocational rehabilitation.ab,ti.  Work adjustment.ab,ti.  Work based.ab,ti.  Work conditioning.ab,ti.  Work disability management.ab,ti.  Work hardening.ab,ti.  Work modification.ab,ti.  Work program.ab,ti.  Work site.ab,ti.  "Work trial*".ab,ti.  Workplace.ab,ti.  "Worksite*".ab,ti.  Workstation adjustment.ab,ti.  motivation.ab,ti.  Occupational Health Promotion.ab,ti.  stress prevention.ab,ti.  cognitive behavioral therapy program.ab,ti. |
