# Supplementary material for: Systematic Review of Workplace Interventions to Support Young Workers’ Safety, Work Environment and Health
Source: J Occup Rehabil. 2024 Apr 30;35(2):215–33. doi: 10.1007/s10926-024-10186-y (PMC12089187; doi:10.1007/s10926-024-10186-y)
Supplement: Supplementary file 2 — Supplementary file2 (DOCX 14 KB) [file 10926_2024_10186_MOESM2_ESM.docx]

# Supplementary material 2: Methodological quality

The table illustrates the questions employed to assess the methodological quality of the selected studies (adapted from Kennedy et al. 2010).

|  | | |
| --- | --- | --- |
| **Question** | **Weight** | |
| 1. Is the research question clearly stated? | | 2 |
| 2. Were comparison group(s) used? | | 3 |
| 3. Was an intervention allocation described adequately? (and was it randomized?) | | 3*2 |
| 4. Was recruitment (or participation) rate reported? | | 2 |
| 5. Were pre-intervention characteristics described? | | 2 |
| 6. Was loss to follow-up (attrition) <35%? | | 2 |
| 7. Did the author examine for important differences between the remaining and drop-out participants after the intervention? | | 2 |
| 8. Was the intervention process adequately described to allow for replication? | | 3 |
| 9. Were the effects of the intervention on some exposure parameters documented? | | 1 |
| 10. Was the participation in the intervention documented? | | 2 |
| 11. Were a relevant outcome measure described at baseline and at follow-up | | 3 |
| 12. Was the length of follow-up three months or greater? | | 2 |
| 13. Was there adjustment for pre-intervention differences (minimum threshold of three important covariates include age, gender and primary outcome at baseline)? | | 3 |
| 14. Were the statistical analyses optimized for the best results? | | 3 |
| 15. Were all participants’ outcomes analyzed by the groups to which they were originally allocated (intention-to-treat analysis)? | | 2 |
| 16. Was there a direct between-group comparison? | | 3 |
