# Supplementary material for: Systematic Review of Workplace Interventions to Support Young Workers’ Safety, Work Environment and Health
Source: J Occup Rehabil. 2024 Apr 30;35(2):215–33. doi: 10.1007/s10926-024-10186-y (PMC12089187; doi:10.1007/s10926-024-10186-y)
Supplement: Supplementary file 3 — Supplementary file3 (DOCX 141 KB) [file 10926_2024_10186_MOESM3_ESM.docx]

# Supplementary material 3: Study Characteristics

The table illustrates characteristics of included studies. The 33 included studies are grouped within 9 different outcome domains and further categorized based on type of intervention by the intervention classification framework. For each study, quality appraisal level (QA) is depicted (H=high, M=medium) based on the guidelines developed by the Institute for Work & Health. See the manuscript for further information.

| **Outcome domain/ type of intervention/ Author (ref)** | **Study design** | **Study population** | **Age (years)** | **Sample size (n)** | **Intervention content** | **Effect of intervention (yes/no)** | **QA** |
| --- | --- | --- | --- | --- | --- | --- | --- |
| **Mental health problems** | | |  |  |  |  |  |
| ***1.1.0.  Attitude and belief*** | | |  |  |  |  |  |
|  | | |  |  |  |  |  |
| Brazier (2022) UK [1] | Randomised controlled trial | Trainee anaesthetists | NA | Intervention: 79 | Text message intervention to reduce negative psychological experiences (burnout specific-ally),as well as increasing positive ones | No, on well-being (p=0,40) | H |
|  |  |  |  | Control: 74 |  | No on burnout (p=0.45) |  |
|  |  |  |  |  |  | No, on meaning (p=0.77) |  |
|  |  |  |  |  |  | No, on value (p=0.99) |  |
|  |  |  |  |  |  |  |  |
| Chen (2017) Taiwan [2] | Randomised controlled trial | Novice nursing practitioner | 22.32 ± 2.66 | Intervention: 16 | An interactive situated and simulated teaching (ISST) pro-gram to reinforce learning incentives, promote participation in nursing activities, and improve care-related concepts in a controlled environment | Yes, on lower stress levels (p= 0.011) | H |
|  |  |  | 22.82 ± 2.61 | Control: 15 |  | Yes, on increased confidence in professional competence (p= 0.026) |  |
|  |  |  |  |  |  | Yes, on superior nursing competency (p= 0.001) |  |
|  |  |  |  |  |  |  |  |
| Guille (2016) United States [3] | Randomised controlled trial | Medical Interns | 25.2 (8.1) | Intervention: 100 | Web-based cognitive behavioural therapy for the prevention of suicidal ideation | Yes, 60% less likely to endorse suicidal ideation during internship year (p=0.03) | H |
|  |  |  |  | Control: 99 |  |  |  |
|  |  |  |  |  |  |  |  |
| Mueller (2018) United States [4] | Other: Randomised intervention study | Doctoral physical therapy students in internship. | 26.8 (3.3, range 20-40) | Intervention: 18 | An online psychology course designed to enhance commitment among caretakers to high quality patient connections with an emphasis on compassion and empathy. | Yes, on work engagement (p=0.040) | H |
|  |  |  |  | Control: 18 |  | Yes, on empathy (p=0.017) |  |
|  |  |  |  |  |  | Yes ^A^, on grit (resilience index score) (p= 0.024) |  |
|  |  |  |  |  |  |  |  |
| Bektas (2018) Turkey [5] | Non-randomised controlled study | Pediatric nursing internship students | 22.44 ± 1.72 | Intervention: 31 | Web-based teaching to improve self-confidence and anxiety levels in the clinical decision‐making process | Yes, on anxiety scores (p=0.002) | M |
|  |  |  | 23.16 ± 1.36 | Control: 30 |  | Yes, on Self-confidence (p=0.000) |  |
|  |  |  |  |  |  |  |  |
| Gunasingam (2015) Australia [6] | Randomised controlled trial | postgraduate year 1 junior doctors | 20-24: 4 | Intervention: 13 | Debriefing sessions with group discussion | No, on burnout scores with debriefing (p>0.05). | M |
|  |  |  | 25-30: 9 20-24: 10  25-30: 6  >30: 2 | Control 18 |  |  |  |
|  |  |  |  |  |  |  |  |
| Muniswamy (2021) India [7] | Randomised controlled trial | Young remotely working IT professionals | 25.32 ± 2.03 | Intervention: 22 | Social media based physical and mental health programme with information regarding physical health (scheduled exercises, desk-based stretches, global PA recommendations, exercise demonstrations, ergonomic advice) and mental health (tips for organised work, work breaks, relaxation techniques) | No, on stress (p>0.05) | M |
|  |  |  | 27.04 ± 4.24 | Control: 26 |  | No, on anxiety (p>0.05) |  |
|  |  |  |  |  |  | No, on depression (p>0.05) |  |
|  |  |  |  |  |  |  |  |
| Petree 2012 United States [8] | Randomised controlled trial | Restaurant Workers | 21.76 | Intervention: 249 | Team Resilience (TR): an inter-active program for stress manage-ment, teamwork, and work-life balance. | Yes, on personal stress (p = .003). | M |
|  |  |  | 21.71 | Control: 236 |  |  |  |
|  |  |  |  |  |  |  |  |
|  |  |  |  |  |  |  |  |
| Pidd 2015 Australia [9] | Randomised controlled trial | Apprentice chefs | 18-24:41 | Intervention: 44 | Psychological wellbeing and sub-stance use intervention for stress management | Yes, on psychological distress (p=0.042; primary outcome) | M |
|  |  |  | >24:30 | Control: 27 |  | No, on life satisfaction (p>0.05) |  |
|  |  |  |  |  |  |  |  |
| Schenkel 2022 Switzerland [10] | Cross-over design | Apprentices from vocational and upper secondary schools and companies. | 17.54 (1.92) | TA-TB: 201 | Mobile phone delivered inter-vention to reduced occupational stress | No, on occupational vigor (p>0.05) | M |
|  |  |  |  | TB-TA: 185 |  | No, on levels of stress (p>0.05) |  |
|  |  |  |  |  |  | No, on work-related exhaustion (p>0.05) |  |
|  |  |  |  |  |  |  |  |
| ***1.2.0.  Behavior based*** | | |  |  |  |  |  |
|  | | |  |  |  |  |  |
| Mohammadi 2019 Iran [11] | Other: Clinical trial | Operating room students during the educational internship | 19.75±0.70 | Intervention: 10 | Educational intervention program provisions included handwashing techniques, sterilization, filling and drop methods, and familiarity with general surgical set | Yes, on situational anxiety scores (p=0.008) | M |
|  |  |  | 19.78±0.97 | Control: 10 |  |  |  |
|  |  |  |  |  |  |  |  |
| Schmidt 2014 France [12] | Randomised controlled trial | 4th year medical students | 22 +/- 1 | Intervention: 74 | Video-based feedback intervention of oral clinical presentations of patients to reduced anxiety | Yes, on anxiety score (p= 0.024) | M |
|  |  |  |  | Control: 68 |  |  |  |
|  |  |  |  |  |  |  |  |
| ***1.4.0 Mental training*** | | |  |  |  |  |  |
|  | | |  |  |  |  |  |
| Lebares 2021 United States [13] | Randomised controlled trial | Surgeons resident trainees | NA | Intervention ESRT 1: 23 | Development of mindfulness meditation skills using culturally acceptable language. | No, on perceived stress (p>0.05) | H |
|  |  |  |  | Intervention ESRT 2: 23 |  |  |  |
|  |  |  |  | Control ESRT 1: 21 |  |  |  |
|  |  |  |  | Control ESRT 2: 22 |  |  |  |
|  |  |  |  |  |  |  |  |
| Koch 2022 Germany [14] | Randomised controlled trial | Police officers in training | 23.35 (4.66) | Intervention: 60 | Yoga-based stress intervention programme | Yes within group effect on perceived stress (reduction in intervention group (p<0.001) and increased in the control group (p<0.001)) | M |
|  |  |  | 23.63 (5.08) | Control: 60 |  |  |  |
|  |  |  |  |  |  |  |  |
| Lebares 2019 United States [15] | Randomised controlled trial | Postgraduate year 1 surgery residents | 29.0 (2.4) | Intervention: 11 | Home based mindfulness based stress reduction intervention | No, on depression (p=0.87) | M |
|  |  |  | 27.4 (2.1) | Control: 10 |  | No, on perceived stress (p=0.19) |  |
|  |  |  |  |  |  | No, on Burnout (p=0.82) |  |
|  |  |  |  |  |  |  |  |
| Loewenthal 2021 United States [16] | Randomised controlled trial | Resident Physicians | 29,3 [26-33] | Intervention: 38 | Yoga-based mind-body intervention | No, on perceived stress (p>0.05) | M |
|  |  |  | 29,1 [27-33] | Control: 18 |  | No, on burnout (p>0.05) |  |
|  |  |  |  |  |  | No, on anxiety (p>0.05) |  |
|  |  |  |  |  |  | No, on well-being (p>0.05) |  |
|  |  |  |  |  |  | No, on work exhaustion (p>0.05) |  |
|  |  |  |  |  |  |  |  |
|  |  |  |  |  |  |  |  |
| Sargolzaei 2021 Iran [17] | Randomised controlled trial | Undergraduate eight-semester OR students who attended their internship program | 22.39 (0,98) | Intervention: 18 | Virtual-Augmented Reality Training on anxiety | Yes, on state anxiety (p=0.001) | H |
|  |  |  | 22.53 (0,62) | Control: 18 |  | Yes, on trait anxiety (p=0.001) |  |
|  |  |  |  |  |  |  |  |
| ***2.1.0. Culture and climate*** | | |  |  |  |  |  |
|  | | |  |  |  |  |  |
| Ripp 2016 United States [18] | Randomised controlled trial | First-Year Internal Medicine Residents | NA | Intervention: 21 | A facilitated group discussion intervention to decrease job burnout | No, on burnout (p>0.05) | M |
|  |  |  |  | Control: 17 |  |  |  |
|  |  |  |  |  |  |  |  |
| ***4.0  Multifaceted*** | | |  |  |  |  |  |
|  | | |  |  |  |  |  |
| Tseng 2021 Taiwan [19] | Other: A quasi-experimental study | (1) New graduates of a two-year baccalaureate program; (2) possessing a nurse certificate; (3) novice workers in a hospital; and (4) consenting to participate in the study. | 22.03 (0.43) | Intervention: 38 | Internship program including planning and supervision, adjustment of the college curriculum, career mentorship and peer support. | No, on occupational stress (p>0.05) | M |
|  |  |  | 22.14 (0.79) | Control: 72 |  |  |  |
|  |  |  |  |  |  |  |  |
| **Psychosocial work factors** | | |  |  |  |  |  |
| ***1.1.0.  Attitude and belief*** | | |  |  |  |  |  |
|  | | |  |  |  |  |  |
| Pidd 2015 Australia [9] | Randomised controlled trial | Apprentice chefs | 18-24:41 >24:30 | Intervention: 44 | Psychological wellbeing and substance use intervention for stress management | No, on social support (p>0.05) | M |
|  |  |  |  | Control: 27 |  |  |  |
|  |  |  |  |  |  |  |  |
|  |  |  |  |  |  |  |  |
|  |  |  |  |  |  |  |  |
| ***1.2.0.  Behavior based*** | | |  |  |  |  |  |
|  | | |  |  |  |  |  |
| Chang 2021 Taiwan [20] | Randomised controlled trial | Senior nursing students who had finished the required professional internship or were undergoing community nursing or psychiatric nursing internship | 21.68 | Intervention: 34 Control: 34 | Sexual harassment knowledge e-book | Yes ^B^, on knowledge of prevention (p=0.004) | M |
|  |  |  |  |  |  | Yes, on sexual harassment coping behaviors (p=0.032) |  |
|  |  |  |  |  |  | Yes ^B^, on knowledge of sexual harassment prevention (p=0.001) |  |
|  |  |  |  |  |  | Yes, on motivation score (p=0.001) |  |
|  |  |  |  |  |  |  |  |
| ***1.4.0 Mental training*** | | |  |  |  |  |  |
|  | | |  |  |  |  |  |
| Loewenthal 2021 United States [16] | Randomised controlled trial | Resident Physicians | 29,3 [26-33] | Intervention: 38 | Yoga-based mind-body intervention | No ^C^, on cynicism (p>0.05) | M |
|  |  |  | 29,1 [27-33] | Control: 18 |  | Not ^C^, on interpersonal disengagement (p>0.05) |  |
|  |  |  |  |  |  | No ^C^, on professional fulfilment (p>0.05) |  |
|  |  |  |  |  |  | Yes, on mindfulness (p=0.004) |  |
| ***2.1.0. Culture and climate*** | | |  |  |  |  |  |
|  | | |  |  |  |  |  |
| Petree 2012 United States [8] | Randomised controlled trial | Restaurant Workers | 21.76 | Intervention: 249 | Team Resilience (TR): an interactive program for stress management, teamwork, and work-life balance. | Yes, on exposure to problem co-workers (p =0.01) | M |
|  |  |  | 21.71 | Control: 236 |  |  |  |
|  |  |  |  |  |  |  |  |
|  |  |  |  |  |  |  |  |
|  |  |  |  |  |  |  |  |
|  |  |  |  |  |  |  |  |
|  |  |  |  |  |  |  |  |
|  |  |  |  |  |  |  |  |
|  |  |  |  |  |  |  |  |
|  |  |  |  |  |  |  |  |
| **Musculoskeletal disorders** | | |  |  |  |  |  |
| ***1.3.0. Physiological modifications*** | | |  |  |  |  |  |
| **1.3.5 Support/compression** | | |  |  |  |  |  |
|  | | |  |  |  |  |  |
| Edgar 2022 New Zealand [21] | Other: Randomised, parallel-group intervention study | Healthy officer trainees | 24 (+/- 6) | Intervention: 27 | Lower-body compression garment use for recovery | No, on soreness (p> 0.05) | M |
|  |  |  |  | Control: 28 |  |  |  |
|  |  |  |  |  |  |  |  |
| Franklyn-Miller 2011 UK [22] | Randomised controlled trial | Military officer trainees | 24.75 [24.68-24.82] | Intervention: 200 Control: 200 | Customized orthoses intervention | Yes, on injuries (P< 0001) | M |
|  |  |  | 24.9 [24.83 - 24.97] |  |  | Yes, on absolute risk reduction of 0.49 from use of the orthoses (P<0001) |  |
|  |  |  |  |  |  |  |  |
| Housler 2017 United States [23] | Randomised controlled trial | US Army Ranger Recruits | NA | SOC+ Procellera: 40 | Bioelectric dressing system, applied topically two to three times per week for 2 weeks to blisters developed | No, on pain (p>0.05) | M |
|  |  |  |  | SOC group: 40 |  |  |  |
|  |  |  |  |  |  |  |  |
| **1.3.1 Mixed physical training** | | |  |  |  |  |  |
|  | | |  |  |  |  |  |
| Borstad 2009 United States [24] | Other: Prospective cohort study | First and second year apprentices from local sheet metal, electrical and plumbing and pipe fitting trades | 26.7 (6.1) | Intervention: 117 Control: 123 | Homebased stretching and strengthening exercise programme | No, on numbers of shoulder pain cases (p>0.05, primary outcome) | M |
|  |  |  |  |  |  | Yes, on relative risk of developing new-onset shoulder pain (RR=1.54) |  |
|  |  |  |  |  |  |  |  |
| ***4.0  Multifaceted*** | | |  |  |  |  |  |
|  | | |  |  |  |  |  |
| Hess 2020 United States [25] | Randomised controlled trial | Masonry apprentices | 28 (7.0) | Intervention E group: 48 | Safety voice for ergonomics program that integrate evidence-based ergonomic training with communication skill training | No, on musculoskeletal symptoms (P>0.180). | M |
|  |  |  | 29 (8.3) | Intervention ESV group: 52 |  |  |  |
|  |  |  | 31 (6.2) | Control: 27 |  |  |  |
|  |  |  |  |  |  |  |  |
|  |  |  |  |  |  |  |  |
|  |  |  |  |  |  |  |  |
| **Ergonomic work factors** | | |  |  |  |  |  |
| ***4.0  Multifaceted*** | | |  |  |  |  |  |
|  | | |  |  |  |  |  |
| Hess 2020 United States [25] | Randomised controlled trial | Masonry apprentices | 28 (7.0) | Intervention E group: 48 | Safety voice for ergonomics program that integrate evidence-based ergonomic training with communication skill training | Yes, on using better body postures (P =0.042) | M |
|  |  |  | 29 (8.3) | Intervention ESV group: 52 |  |  |  |
|  |  |  | 31 (6.2) | Control: 27 |  |  |  |
|  |  |  |  |  |  |  |  |
| Muniswamy 2021 India [7] | Randomised controlled trial | Young remotely working IT professionals | 25.32 ± 2.03 | Intervention: 22 | Social media based physical and mental health programme with information regarding physical health (scheduled exercises, desk-based stretches, global PA recommendations, exercise demonstrations, ergonomic advice) and mental health (tips for organised work, work breaks, relaxation techniques) | No, on sitting time during work (p>0.05) | M |
|  |  |  | 27.04 ± 4.24 | Control: 26 |  |  |  |
|  |  |  |  |  |  |  |  |
| **Injuries** | | |  |  |  |  |  |
| ***4.4 Multifaceted, across level*** | | |  |  |  |  |  |
|  | | |  |  |  |  |  |
| Nielsen 2019 Denmark [26] | Non-randomised controlled study | Apprentice in small construction companies | NA | Intervention: 20 companies | Safety training targeting owner-managers: both active work environment factors (checklist approach) and motivational factors (assistance with mandatory systematic health and safety obligations, access to promotional materials and an option to get a free visit from a health and safety advisor) | No, on injuries (p = 0.25) | M |
|  |  |  |  | Control: 22 companies |  |  |  |
|  |  |  |  |  |  |  |  |
|  |  |  |  |  |  |  |  |
|  |  |  |  |  |  |  |  |
|  |  |  |  |  |  |  |  |
| ***1.3.0. Physiological modifications*** | | |  |  |  |  |  |
| **1.3.5 support/compression** | | |  |  |  |  |  |
|  | | |  |  |  |  |  |
| Franklyn-Miller 201 UK [22] | Randomised controlled trial | Military officer trainees | 24.8 [24.7-24.8] 24.9 [24.8-25.0] | Intervention: 200 Control: 200 | Customized orthoses intervention | Yes, on fever injuries in the orthotic intervention group (P< 0001) | M |
|  |  |  |  |  |  | Yes, on absolute risk reduction from use of the orthoses (P<0001) |  |
|  |  |  |  |  |  |  |  |
| Housler 2017 United States [23] | Randomised controlled trial | US Army Ranger Recruits | NA | SOC+ Procellera: 40 | Bioelectric dressing system, applied topically two to three times per week for 2 weeks to blisters developed | No, on wound healing (p>0.05) | M |
|  |  |  |  | SOC group: 40 |  |  |  |
|  |  |  |  |  |  |  |  |
| **Hand eczema** | | |  |  |  |  |  |
| ***4.1 Multifaceted Individual level*** | | |  |  |  |  |  |
|  | | |  |  |  |  |  |
| Bregnhoj 2012 Denmark [27] | Other: Clinically controlled, prospective interventions study | Hairdressing apprentices | 17.5 | Intervention: 301 | A 2-day course of special training in skin physiology, allergy and eczema, prevention of hand eczema among hairdressers and optimisation of workplace procedure. | Yes, on experienced hand eczema (p= 0.04)  Yes, on the use of gloves for wet work (p=0.004) | M |
|  |  |  |  | Control: 201 |  |  |  |
|  |  |  |  |  |  |  |  |
|  |  |  |  |  |  |  |  |
| Moldovan 2021 Romania [28] | Other: Interventional, multicenter, prospective, controlled study | Nurse Apprentice | 26.01 (19-52) 25.81 (19-46) | Intervention: 97 Control: 42 | Short training program regarding the prevention of hand eczema (skin biology and eczema, the proper use of soap, disinfectants`,  and emollients) and provision of emollients and soaps to be used during work. | Yes ^D^, on objectively measured cutaneous parameters: stratum corneum hydration (p=0.006) and trans epidermal water loss (p<0.001) | M |
|  |  |  |  |  |  | No, on subjective hand eczema severity index (p=0.806) |  |
|  |  |  |  |  |  |  |  |
|  |  |  |  |  |  |  |  |
| Reich 2020 Germany [29] | Non-randomised controlled study | Metal work apprentices (office work apprentices served as controls) | 18.37 ± 3.27 | MW Intervention: 131 | Training on causes and prevention of work-related hand eczema consisting of interactive and dialogue-oriented presentations combined with hands-on practical exercises on correct use of protective gloves, barrier creams, and skin care products. | Yes, on incidence of hand eczema (p< .01) | M |
|  |  |  | 19.21 ± 3.34 | MW Control: 172 |  |  |  |
|  |  |  | 20.58 ± 3.09 | OW Control: 118 |  |  |  |
|  |  |  |  |  |  |  |  |
| Wilke 2018 Germany [30] | Other: Prospective Longitudinal Study | Apprentices of health-related (nursing assistants, geriatric nurses, and doctors assistants) and non-health-related professions (metalworkers, cutting machine operators, and motorcar mechanics)) | 20.40 20.20 | Intervention: 140 Control: 134 | One time skin protection seminar on occupational skin diseases and appropriate skin protection behaviour with a focus on collaboration of participants and the involvement of apprentices in hands-on skin protection experiments. | No ^E^, on prevalence of skin changes at the elbows flexures and at the wrists (p>0.05) | M |
|  |  |  |  |  |  | Yes, on knowledge of occupational skin diseases p<0.001) |  |
|  |  |  |  |  |  |  |  |
| **Safety** | | |  |  |  |  |  |
| ***1.1.3  Teaching and education*** | | |  |  |  |  |  |
|  | | |  |  |  |  |  |
| Rohlman 2016 United States [31] | Randomised controlled trial | Younger workers hired for summer work at a large parks and recreation organization | 17.7 (0.2) 18.2 (0.2) | Intervention: 128 Control: 127 | Electronically delivered health and Safety training and talking safety curriculum, health promotion (nutrition, hydration, sleep, and substance abuse), and effective communication in the workplace. | Yes, on increased safety and health knowledge (p<0.001) | M |
|  |  |  |  |  |  | Yes, on safety behavior (p=0.009) |  |
|  |  |  |  |  |  | Yes, on safety attitude (p=0.015) |  |
|  |  |  |  |  |  |  |  |
|  |  |  |  |  |  |  |  |
|  |  |  |  |  |  |  |  |
|  |  |  |  |  |  |  |  |
|  |  |  |  |  |  |  |  |
|  |  |  |  |  |  |  |  |
|  |  |  |  |  |  |  |  |
| ***4.1 Multifaceted Individual level*** | | |  |  |  |  |  |
|  | | |  |  |  |  |  |
| Hess 2020 United States [25] | Randomised controlled trial | Masonry apprentices | 28 (7.0) | Intervention E group: 48 | Safety voice for ergonomics program that integrate evidence-based ergonomic training with communication skill training | Yes ^F^, on safety voice use (p=0.003) | M |
|  |  |  | 29 (8.3) | Intervention ESV group: 52 |  | No, on safety participation, safety compliance |  |
|  |  |  | 31 (6.2) | Control: 27 |  |  |  |
|  |  |  |  |  |  |  |  |
|  |  |  |  |  |  |  |  |
|  |  |  |  |  |  |  |  |
| ***4.4 Multifaceted, across level*** | | |  |  |  |  |  |
| Nielsen 2019 Denmark [26] | Non-randomised controlled study | Apprentice in small construction companies | NA | Intervention: 20 companies | Safety training targeting owner-managers: both active work environment factors (checklist approach) and motivational factors (assistance with mandatory systematic health and safety obligations, access to promotional materials and an option to get a free visit from a health and safety advisor) | No, on safety climate for apprentices (p>0.05), but improvement for journeymen in safety climate items: convenience violations (p = 0.04) and company risk acceptance (p = 0.03) | M |
|  |  |  |  | Control: 22 companies |  |  |  |
|  |  |  |  |  |  |  |  |
| **Health and physical activity** | | |  |  |  |  |  |
| ***1.1.0.  Attitude and belief*** | | |  |  |  |  |  |
|  | | |  |  |  |  |  |
| Brazier 2022 UK [1] | Randomised controlled trial | Trainee anaesthetists | NA | Intervention: 79 | Text message intervention to reduce negative psychological experiences (burnout specifically),as well as increasing positive ones | No, on sick days (p=0.56) | H |
|  |  |  |  | Control: 74 |  | No, on consideration of career break (p=0.24). |  |
|  |  |  |  |  |  |  |  |
|  |  |  |  |  |  |  |  |
|  |  |  |  |  |  |  |  |
|  |  |  |  |  |  |  |  |
|  |  |  |  |  |  |  |  |
|  |  |  |  |  |  |  |  |
|  |  |  |  |  |  |  |  |
| ***1.4.0 Mental training*** | | |  |  |  |  |  |
|  | | |  |  |  |  |  |
| Koch 2022 Germany [14] | Randomised controlled trial | Police officers in training | 23.35 (4.66) | Intervention: 60 | yoga-based stress intervention programme | Yes, within group reduction in DBP in the right arm of 1.34 mmHg (p=0.007) whereas the control group showed a slight increase in DBP in both arms (right arm p<0.001, left arm p=0.030) | M |
|  |  |  | 23.63 (5.08) | Control: 60 |  |  |  |
|  |  |  |  |  |  |  |  |
| ***4.0  Multifaceted*** | | |  |  |  |  |  |
|  | | |  |  |  |  |  |
| Grüne 2022 Germany [32] | Other: Mixed: Non-randomized controlled trials | Automotive mechatronics and nursing care institutions | 18.39 (3.12) | Intervention G-A: 23 | A tutoring system where apprentices act as PA prompter for their colleagues: co-created physical activity interventions in vocational education and training (covering PA and health in theory and practice) | No, on physical activity (p>0.05) | M |
|  |  |  |  | Control G-A: 37 |  | No, on physical activity-related health competence (p>0.05) |  |
|  |  |  |  | Intervention G-N: 17 Control G-N: 34 |  |  |  |
|  |  |  |  |  |  |  |  |
|  |  |  |  |  |  |  |  |
| Muniswamy 2021  India [7] | Randomised controlled trial | Young remotely working IT professionals | 25.32 ± 2.03 27.04 ± 4.24 | Intervention: 22 Control: 26 | Social media based physical and mental health programme with information regarding physical health (scheduled exercises, desk-based stretches, global PA recommendations, exercise demonstrations, ergonomic advice) and mental health (tips for organized work, work breaks, relaxation techniques) | Yes, on sitting time during non-working days (p<0.004) | M |
|  |  |  |  |  |  |  |  |
|  |  |  |  |  |  |  |  |
|  |  |  |  |  |  |  |  |
|  |  |  |  |  |  |  |  |
|  |  |  |  |  |  |  |  |
|  |  |  |  |  |  |  |  |
|  |  |  |  |  |  |  |  |
|  |  |  |  |  |  |  |  |
|  |  |  |  |  |  |  |  |
| **Physical capacity** | | |  |  |  |  |  |
| ***1.3.0. Physiological modifications*** | | |  |  |  |  |  |
| **1.3.1 Mixed physical training** | | |  |  |  |  |  |
|  | | |  |  |  |  |  |
| Dijksma 2020 The Netherlands [33] | Randomized controlled trials | A special infantry unit of the Netherlands Armed Forces | 20 (19-21) | Intervention: 26 | A physical fitness training program focused on functional training to improve mobility, power, agility, strength, and cardiovascular endurance | No, on aerobic endurance were observed (p>0.05) | M |
|  |  |  |  | Control: 23 |  |  |  |
|  |  |  |  |  |  |  |  |
|  |  |  |  |  |  |  |  |
|  |  |  |  |  |  |  |  |
| **1.3.5 Support/compression** | | |  |  |  |  |  |
|  | | |  |  |  |  |  |
| Edgar 2022  New Zealand [21] | Other: Randomised, parallel-group intervention study | Healthy officer trainees | 24 (+/- 6) | Intervention: 27 | lower-body compression garment use for recovery | No, on performance measures (p> 0.05). | M |
|  |  |  |  | Control: 28 |  |  |  |
|  |  |  |  |  |  |  |  |
|  |  |  |  |  |  |  |  |
| ***4.0  Multifaceted*** | | |  |  |  |  |  |
|  | | |  |  |  |  |  |
| Muniswamy 2021  India [7] | Randomised controlled trial | Young remotely working IT professionals | 25.32 ± 2.03 | Intervention: 22 | Social media based physical and mental health programme with information regarding physical health (scheduled exercises, desk-based stretches, global PA recommendations, exercise demonstrations, ergonomic advice) and mental health (tips for organised work, work breaks, relaxation techniques) | No, on maximal aerobic capacity (p=0.993) | M |
|  |  |  | 27.04 ± 4.24 | Control: 26 |  |  |  |

NA = not available/applicable

Age: Illustrated as mean, median, percent, or range

^A^ = No effect on grit from T2 to T3 p=0.245

^B^ = No effect at 2 weeks post-test

^C^ = Overall no effect

^D^ = Effect on the objectively measured but not subjectively measured outcome. Based on the aim and inclusion criteria the overall effect is categorized as ‘yes’.

^E^ = Categorized as no overall effect

^F^  = Categorized as an overall effect

1. Brazier A, Larson E, Xu Y, Judah G, Egan M, Burd H, et al. 'Dear Doctor': a randomised controlled trial of a text message intervention to reduce burnout in trainee anaesthetists. Anaesthesia. 2022;77(4):405-15.

2. Chen SH, Chen SC, Lee SC, Chang YL, Yeh KY. Impact of interactive situated and simulated teaching program on novice nursing practitioners' clinical competence, confidence, and stress. Nurse education today. 2017;55:11-6.

3. Guille C, Zhao Z, Krystal J, Nichols B, Brady K, Sen S. Web-Based Cognitive Behavioral Therapy Intervention for the Prevention of Suicidal Ideation in Medical Interns: A Randomized Clinical Trial. Missouri medicine. 2016;113(1):19.

4. Mueller K, Prins R, de Heer HD. An online intervention increases empathy, resilience, and work engagement among physical therapy students. Journal of allied health. 2018;47(3):196-203.

5. Bektas I, Yardimci F. The effect of web-based education on the self-confidence and anxiety levels of paediatric nursing interns in the clinical decision-making process. Journal of Computer Assisted Learning. 2018;34(6):899-906.

6. Gunasingam N, Burns K, Edwards J, Dinh M, Walton M. Reducing stress and burnout in junior doctors: the impact of debriefing sessions. Postgraduate medical journal. 2015;91(1074):182-7.

7. Muniswamy P, Gorhe V, Parashivakumar L, Chandrasekaran B. Short-term effects of a social media-based intervention on the physical and mental health of remotely working young software professionals: A randomised controlled trial. Applied psychology Health and well-being. 2021.

8. Petree RD, Broome KM, Bennett JB. Exploring and Reducing Stress in Young Restaurant Workers: Results of a Randomized Field Trial. American Journal of Health Promotion. 2012;26(4):217-24.

9. Pidd K, Roche A, Fischer J. A recipe for good mental health: A pilot randomised controlled trial of a psychological wellbeing and substance use intervention targeting young chefs. Drugs: Education, Prevention & Policy. 2015;22(4):352-61.

10. Schenkel K, Haug S, Castro RP, Lüscher J, Scholz U, Schaub MP, et al. One SMS a day keeps the stress away? A just-in-time planning intervention to reduce occupational stress among apprentices. Applied psychology Health and well-being. 2022.

11. Mohammadi G, Tourdeh M, Ebrahimian A. Effect of simulation-based training method on the psychological health promotion in operating room students during the educational internship. Journal of education and health promotion. 2019;8:172.

12. Schmidt M, Freund Y, Alves M, Monsel A, Labbe V, Darnal E, et al. Video-based feedback of oral clinical presentations reduces the anxiety of ICU medical students: a multicentre, prospective, randomized study. BMC medical education. 2014;14:103.

13. Lebares CC, Coaston TN, Delucchi KL, Guvva EV, Shen WT, Staffaroni AM, et al. Enhanced Stress Resilience Training in Surgeons: Iterative Adaptation and Biopsychosocial Effects in 2 Small Randomized Trials. Annals of surgery. 2021;273(3):424-32.

14. Koch S, Esch T, Werdecker L. Effects of a Yoga-Based Stress Intervention Program on the Blood Pressure of Young Police Officers: A Randomized Controlled Trial. Journal of integrative and complementary medicine. 2022;28(3):234-40.

15. Lebares CC, Guvva EV, Olaru M, Sugrue LP, Staffaroni AM, Delucchi KL, et al. Efficacy of Mindfulness-Based Cognitive Training in Surgery: Additional Analysis of the Mindful Surgeon Pilot Randomized Clinical Trial. JAMA network open. 2019;2(5):e194108.

16. Loewenthal J, Dyer NL, Lipsyc-Sharf M, Borden S, Mehta DH, Dusek JA, et al. Evaluation of a Yoga-Based Mind-Body Intervention for Resident Physicians: A Randomized Clinical Trial. Global advances in health and medicine. 2021;10:21649561211001038.

17. Sargolzaei F, Omid A, Mirmohammad-Sadeghi M, Ghadami A. The effects of virtual-augmented reality training on anxiety among operating room students attending coronary artery bypass graft surgery. Nursing and Midwifery Studies. 2021;10(4):229-35.

18. Ripp JA, Fallar R, Korenstein D. A Randomized Controlled Trial to Decrease Job Burnout in First-Year Internal Medicine Residents Using a Facilitated Discussion Group Intervention. Journal of graduate medical education. 2016;8(2):256-9.

19. Tseng MY, Hwang SL. "SURVIVAL" intervention program effects on student nurses' transition to staff nurses: A quasi-experimental study. Nurse education in practice. 2021;56:103184.

20. Chang TS, Teng YK, Chien SY, Tzeng YL. Use of an interactive multimedia e-book to improve nursing students'' sexual harassment prevention knowledge, prevention strategies, coping behavior, and learning motivation: A randomized controlled study. Nurse education today. 2021;105:104883.

21. Edgar DT, Beaven CM, Gill ND, Driller MW. Under Pressure: The Chronic Effects of Lower-Body Compression Garment Use during a 6-Week Military Training Course. International journal of environmental research and public health. 2022;19(7).

22. Franklyn-Miller A, Wilson C, Bilzon J, McCrory P. Foot orthoses in the prevention of injury in initial military training: a randomized controlled trial. The American journal of sports medicine. 2011;39(1):30-7.

23. Housler GJ, Cross S, Marcel V, Kennedy DO, Husband M, Register A, et al. A Prospective Randomized Controlled Two-Arm Clinical Study Evaluating the Efficacy of a Bioelectric Dressing System for Blister Management in US Army Ranger Recruits. Journal of special operations medicine : a peer reviewed journal for SOF medical professionals. 2017;17(2):49-58.

24. Borstad JD, Buetow B, Deppe E, Kyllonen J, Liekhus M, Cieminski CJ, et al. A longitudinal analysis of the effects of a preventive exercise programme on the factors that predict shoulder pain in construction apprentices. Ergonomics. 2009;52(2):232-44.

25. Hess JA, Kincl L, Weeks DL, Vaughan A, Anton D. Safety Voice for Ergonomics (SAVE): Evaluation of a masonry apprenticeship training program. Applied ergonomics. 2020;86.

26. Nielsen KJ, Grytnes R, Dyreborg J. Pilot test of a tailored intervention to improve apprentice safety in small construction companies. Safety Science. 2019;117:305-13.

27. Bregnhoj A, Menne T, Johansen JD, Sosted H. Prevention of hand eczema among Danish hairdressing apprentices: an intervention study. Occupational and environmental medicine. 2012;69(5):310-6.

28. Moldovan HR, Manole I, Suru A, Butacu AI, Tatu AL, Lupu A, et al. Prevention of Hand Eczema among Nurse Apprentice (PREVEDERM): An Interventional Study. Annals of work exposures and health. 2021;65(2):167-75.

29. Reich A, Wilke A, Gediga G, Baurecht H, Rodríguez E, Jakasa I, et al. Health education decreases incidence of hand eczema in metal work apprentices: Results of a controlled intervention study. Contact dermatitis. 2020;82(6):350-60.

30. Wilke A, Brans R, Nordheider K, Braumann A, Hubner A, Sonsmann FK, et al. Skin Protection Seminars to Prevent Occupational Skin Diseases: Results of a Prospective Longitudinal Study in Apprentices of High-risk Professions. Safety and health at work. 2018;9(4):398-407.

31. Rohlman DS, Parish M, Elliot DL, Hanson G, Perrin N. Addressing Younger Workers' Needs: The Promoting U through Safety and Health (PUSH) Trial Outcomes. Healthcare (Basel, Switzerland). 2016;4(3).

32. Grüne E, Popp J, Carl J, Semrau J, Pfeifer K. Examining the sustainability and effectiveness of co-created physical activity interventions in vocational education and training: a multimethod evaluation. BMC public health. 2022;22(1):765.

33. Dijksma I, Zimmermann WO, Bovens D, Lucas C, Stuiver MM. Despite an improved aerobic endurance, still high attrition rates in initially low-fit recruits-results of a randomised controlled trial. Contemporary clinical trials communications. 2020;20:100679.
